# Local selection in the presence of high levels of gene flow: Evidence of heterogeneous insecticide selection pressure across Ugandan *Culex quinquefasciatus* populations

**DOI:** 10.1371/journal.pntd.0005917

**Published:** 2017-10-03

**Authors:** Walter Fabricio Silva Martins, Craig Stephen Wilding, Keith Steen, Henry Mawejje, Tiago Rodrigues Antão, Martin James Donnelly

**Affiliations:** 1 Department of Vector Biology, Liverpool School of Tropical Medicine, Liverpool, United Kingdom; 2 Departamento de Biologia, Universidade Estadual da Paraíba, Campina Grande, Brasil; 3 School of Natural Sciences and Psychology, Liverpool John Moores University, Liverpool, United Kingdom; 4 IDRC, Kampala, Uganda; 5 Division of Biological Science, University of Montana, Missoula, United States of America; 6 Malaria Programme, Wellcome Trust Sanger Institute, Cambridge, United Kingdom; North Carolina State University, UNITED STATES

## Abstract

**Background:**

*Culex quinquefasciatus* collected in Uganda, where no vector control interventions directly targeting this species have been conducted, was used as a model to determine if it is possible to detect heterogeneities in selection pressure driven by insecticide application targeting other insect species.

**Methodology/Principal findings:**

Population genetic structure was assessed through microsatellite analysis, and the impact of insecticide pressure by genotyping two target-site mutations, *Vgsc*-*1014F* of the voltage-gated sodium channel target of pyrethroid and DDT insecticides, and *Ace1*-*119S* of the acetylcholinesterase gene, target of carbamate and organophosphate insecticides. No significant differences in genetic diversity were observed among populations by microsatellite markers with *H*_E_ ranging from 0.597 to 0.612 and low, but significant, genetic differentiation among populations (*F*_*ST*_ = 0.019, *P* = 0.001). By contrast, the insecticide-resistance markers display heterogeneous allelic distributions with significant differences detected between Central Ugandan (urban) populations relative to Eastern and Southwestern (rural) populations. In the central region, a frequency of 62% for *Vgsc*-*1014F*, and 32% for the *Ace1-119S* resistant allele were observed. Conversely, in both Eastern and Southwestern regions the *Vgsc*-*1014F* alleles were close to fixation, whilst *Ace1-119S* allele frequency was 12% (although frequencies may be underestimated due to copy number variation at both loci).

**Conclusions/Significance:**

Taken together, the microsatellite and both insecticide resistance target-site markers provide evidence that in the face of intense gene flow among populations, disjunction in resistance frequencies arise due to intense local selection pressures despite an absence of insecticidal control interventions targeting *Culex*.

## Author summary

*Culex quinquefasciatus* is a primary vector of arboviruses such as West Nile virus (WNV) and St. Louis encephalitis virus (SLEV) in temperate regions, and in many tropical/sub-tropical regions is implicated in the transmission of lymphatic filariasis (LF). Insecticide-based approaches are one of the most important interventions to mitigate disease burden; nevertheless, increased resistance of vectors to insecticides poses a challenge for sustainability and effectiveness of both current and future vector control interventions. Here, *Cx*. *quinquefasciatus* collected across a transect from Eastern to South Western Uganda were utilized to infer the likely pattern of the evolution of insecticide resistance among populations through a combination of putative selective loci (the target-site mutations *Vgsc*-*1014F* and *Ace1-119S*) and 26 neutral microsatellite markers. Taken together, the results yielded by these markers provide evidence that Ugandan *Cx*. *quinquefasciatus* mosquitoes are under heterogeneous selection pressure imposed by insecticides from distinct classes.

## Introduction

Contemporary evolution of insecticide resistance in mosquitoes, which threatens the effectiveness of insecticide-based approaches to reduce the burden of vector-borne disease in endemic regions, results from a complex interaction of biological traits (e.g. genetic mechanisms of resistance) and operational implementation of control programs (e.g. choice of insecticide class and selection strength see [1, 2]).

Target-site mutations in either the acetylcholinesterase gene (*Ace-1*) or the voltage-gated sodium channel (*Vgsc*) gene, as well as metabolic resistance mediated by upregulation of, or mutations in, detoxification gene families such as the cytochrome P450 (P450s), esterases and glutathione S-transferases (GSTs) are the most well studied genetic mechanisms underpinning resistance [3–5]. Pyrethroids or DDT bind to the VGSC neuronal ion channel resulting in the channel pore being unable to close, so causing repetitive nerve firing, paralysis and death [6]. A number of resistance mutations have been identified in this gene in a range of insect species with an L to F change at codon 1014 frequently identified [7]. By contrast, carbamate and organophosphate insecticides target the enzyme acetylcholinesterase (AChE) at cholinergic synapses, blocking transmission of nerve impulses by inhibition of AChE. A G to S mutation at codon 119 (*Torpedo* numbering system) of the *Ace-1* gene results in resistance and is widespread in organophosphate and carbamate selected populations [8, 9]. In *Culex* and other vectors and pest insects the intense use of insecticides has been associated with sharp increases of *Vgsc*-*1014F* –also called *kdr* (knock-down resistance) [10–12] and an increased frequency of the *Ace1*-*119S* resistant allele.

Despite the recognized role of such target-site mutations in the adaptability of mosquitoes under insecticide selection, many studies have also shown that such resistant-associated mutations can have a range of deleterious effects and fitness disadvantages (see [13]). In *Culex* and other resistant insect populations where such fitness detriments are seen, the directional selection of adaptive target-site mutations has been linked with an increase in gene copy number. The most well documented example of this is for the duplication of *Ace*-*1*, which has been reported in diverse arthropod species such as *Aphis gossypii*, *Tetranychus evansi*, *Anopheles gambiae* as well as in *Culex* from a range of geographic regions [14–17]. Many studies have proposed that duplication of *Ace*-*1* acts as a compensatory adaptive mechanism to restore the disrupted function of the nervous system driven by the resistant allele so reducing the fitness costs [18–20]. Additionally, duplication of the *Vgsc* gene was also recently reported in both *Cx*. *quinquefasciatus* and *Aedes aegypti*, indicating a likely adaptive role of gene duplication in the evolution of resistance to pyrethroids [21, 22].

In addition to the underlying genomic basis of resistance, the evolutionary pattern of insecticide resistance can also be influenced by the heterogeneity of environmental selection pressures encountered by mosquito populations. For example, mosquitoes from urban and rural settlements could experience contrasting patterns of selection imposed by direct or indirect vector control interventions, and/or the indirect impact of pesticides and environmental pollution encountered by vectors in rural settings [23]. From a vector control perspective, developing selective control approaches targeting only the primary species of interest is challenging since in most endemic regions, species with roles in vector-borne disease transmission can be co-endemic, heightening the risk of insecticide selective pressure on non-target vector species.

On the African continent, lymphatic filariasis (LF) is predominantly transmitted by *Anopheles* mosquitoes in rural areas, while in urban and coastal areas of East Africa *Culex* species are the main vector [24]. In Uganda, a country with high rates of malaria transmission and LF prevalence, *Anopheles* mosquitoes are incriminated as the primary vector of both diseases [25, 26]. Therefore, vector control operations using insecticide-based approaches, either through insecticide treated nets (ITN) or indoor residual spraying (IRS), chiefly target *Anopheles*. Whilst there is no state vector control programme targeting *Culex* populations across Uganda, insecticide resistance may be indirectly selected through the use of insecticides for the control of *Anopheles*, other vectors or pest species [23, 27].

In this study, we used collections of *Cx*. *quinquefasciatus* from Uganda as a model to determine if it is possible to infer heterogeneities in insecticide selection on non-target species through combined study of putatively selected resistant alleles (*Vgsc*-*1014F* and *Ace1*-*119S*) and neutral markers (microsatellites). To this end, microsatellite markers were developed and utilized to detect likely influence of geographic and colonization events on *Cx*. *quinquefasciatus* population diversity and structure [28, 29]. By contrast, changes in the pattern of resistant alleles (*Vgsc*-*1014F* and *Ace1*-*119S*) was used a proxy of the strength of insecticidal selection across Uganda.

## Materials and methods

### Ethics statement

Ethical approval was obtained from the Makerere University School of Medicine Research and Ethics Committee. This was an experimental setting for research purposes and was not linked to a malaria control program. Adult household representatives gave written informed consent with the Ethics Statement before entry into their houses for mosquito aspiration. This study used solely mosquito samples, while no human participants were involved.

### Area of study and mosquito collection

**Fig 1:**
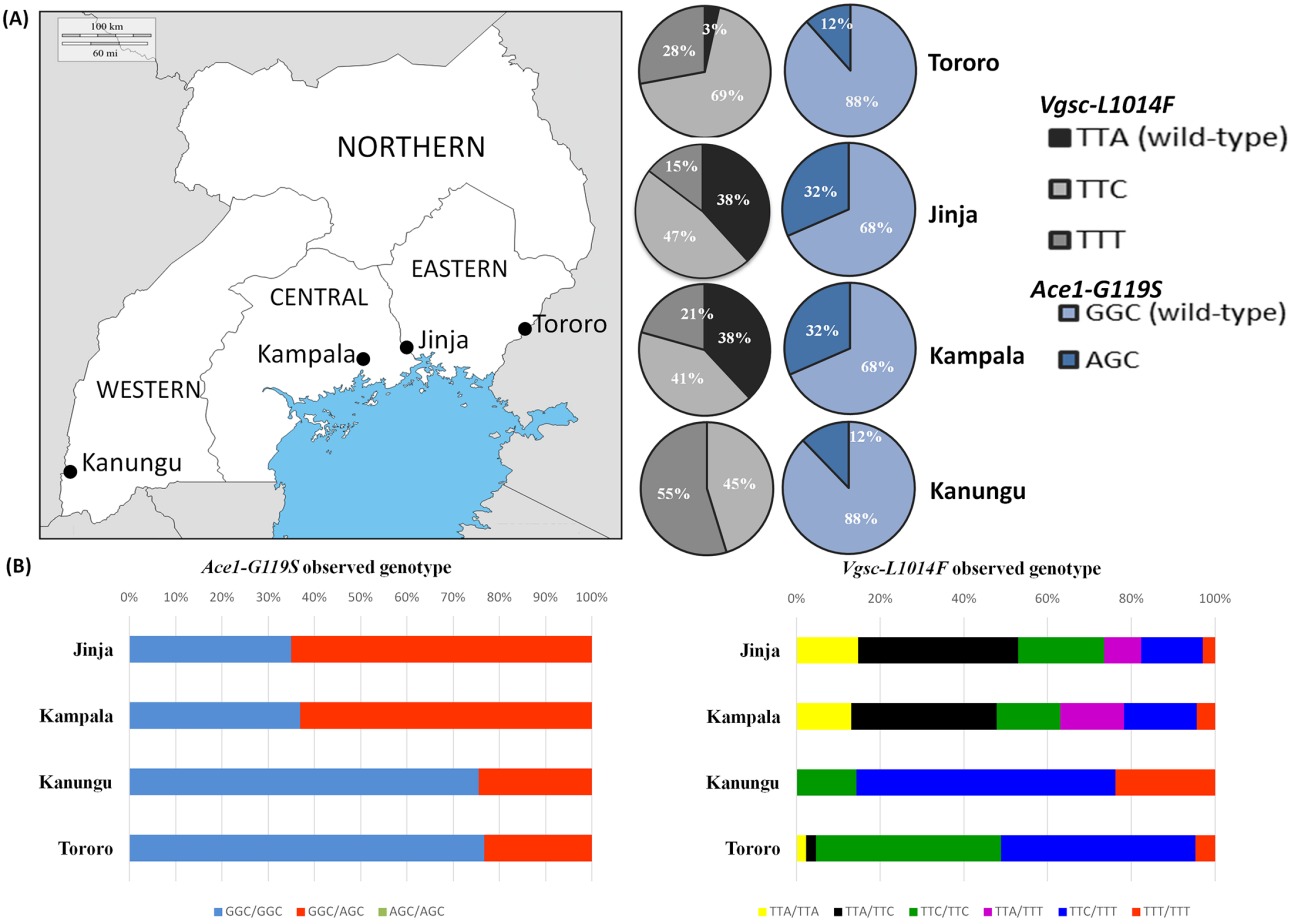
Geographic distribution of insecticide target-site mutations *Ace1-G119S* and *Vgsc*-*L1014F* in field-collected Ugandan *Cx*. *quinquefasciatus* mosquitoes. (A) Geographic location of mosquito collections and frequency of target-site mutations. Pie charts depict the relative frequency of *Ace1*-*G119S* and *Vgsc*-*L1014F* mutations. Image sourced from https://commons.wikimedia.org/wiki/Uganda via the United States Central Intelligence Agency (https://www.cia.gov/library/publications/the-world-factbook/geos/ug.html). The image is originally published under the CC0 public domain license (B) Bar charts show the genotypic frequency of target-site loci.

*Cx*. *quinquefasciatus* mosquitoes were collected across a transect from Eastern to Southwestern Uganda, with collection points located in four districts (Fig 1A, S1 and S2 Tables). Collection points were located in Tororo and Kanungu districts (located in the Eastern and Western administrative regions) with populations of 517,000 and 252,000 people respectively and where more than 80% of dwellings are rural, and in Jinja and Kampala (Central region) with populations of 76,000 and 1.5 million people respectively [30, 31]. Samples were from a peri-urban area in Jinja (63% of houses in rural areas) and in the urban centre of Kampala.

From each collection site adult male and female mosquitoes were collected by aspiration from inside 10–15 houses (up to 10 mosquitoes per house) between July and August 2012. Then, a sub-set of the samples (S2 Table) were used for microsatellite and target-site mutation genotyping as described below.

Collected mosquitoes are representative of three Ugandan Demographic and Health Survey (UDHS) regions; Central, South-western and Mid-eastern with similar levels of insecticide-treated net (ITNs) coverage i.e. percentage of householders with at least one ITN ranging from 56 to 58.6% reported in 2011 [30] (S1 Table). However, the insecticide usage pattern in Uganda has changed rapidly in recent years, therefore the data provided (S1 Table) reflects very recent use and it does not absolutely reflect the last 5–10 years when resistance may have developed [32, 33]. Therefore, the ITN and IRS figures from 2011 for each UDHS are used herein as a proxy of likely selection strength among the collection sites, although they may not represent precisely the sources and strength of selection in each location. Indeed, recently Abeku *et al*. [34] have demonstrated that the effect of ITN usage on *Kdr* allele frequency is impacted by annual rainfall.

Across Uganda, two main pyrethroid based ITNs have been distributed by the government; Permanet 2.0 (polyester coated with deltamethrin) and Olyset Nets (polyethylene with permethrin incorporated). Additionally, a distinct level of ITN usage and Indoor Residual Spraying (IRS) is reported throughout the UDHS regions from where samples were collected (S1 Table). While major IRS programmes have not been reported in Jinja or Tororo, in Kanungu, approximately 45,000 households covering a population of 190,000 were sprayed with λ-cyhalothrin in 2007 as part of the malaria control programme [35]. In Kampala, a programme of environmental management was undertaken in the early 2000s (http://health.go.ug/programs/national-malaria-control-program) but no sustained insecticidal vector control has been used.

### DNA isolation and species identification

Genomic DNA from individual mosquitoes was isolated using a DNeasy kit (Qiagen) following the manufacturer’s recommendations. All samples were confirmed as *Cx*. *quinquefasciatus* by a diagnostic PCR assay which types diagnostic length polymorphisms in the second intron of the acetylcholinesterase-2 gene to differentiate *Cx*. *quinquefasciatus* from *Cx*. *pipiens* [36].

### Allelic discrimination assays of insecticide target-site mutations

To facilitate design of target-site mutation genotyping assays, partial genomic fragments spanning the location of the *G119S* mutation in the *Ace-1* gene and the *L1014F* mutation in the *Vgsc* gene were amplified from genomic DNA isolated from mosquitoes collected from the four populations. A TaqMan assay targeting the *Ace-1* gene was designed to genotype the SNP (GGC/AGC) in the first base of the codon at position 119, while a *Vgsc*-pyrosequencing assay to type the *L1014F* mutation in exon 20 [6] of the *Vgsc* gene was developed to detect two synonymous resistant alleles (TTC and TTT) and the wild-type allele (TTA) (S1 Methods). We chose the pyrosequencing method for genotyping *Vgsc*-*L1014F* instead of applying the widely used Taqman assay due to the existence of three alleles at this codon, which limits the use of Taqman for genotyping in a single reaction. Further details for PCR amplification of the partial fragment of both genes, as well as the target-site assay primer and probe sequences and reaction conditions are provided in the S1 Methods.

In the studied populations, genotyping of both *Ace*-*1* and the *Vgsc* indicated the presence of gene duplication. Such gene duplications hinder calculation of many population-genetic statistics such as allele frequencies, linkage disequilibrium (LD) and population differentiation especially when there is polymorphism for copy number. Indeed, the absence of straightforward genotyping and data analysis methods limited our ability to address further the implications of the gene duplication in the evolution of resistance, with similar drawbacks also reported in other studies [15, 37]. Due to the limitations of the genotyping methods (e.g. it is not possible to differentiate the genotypes RRSS from RRRS), our analysis is based solely on the presence/absence of resistant and susceptible alleles as applied to a single copy gene. This assumption could result in an underestimation of insecticide associated-resistant alleles within the studied populations whilst still allowing us to infer a proxy of increased resistance allele frequency in a population under selection pressure.

### Detection of *Ace-1* gene duplication by haplotype diversity

The absence of *Ace-1* homozygous resistant genotypes (AGC/AGC) observed through Taqman (see Results) indicated a likely gene duplication of *Ace-1* in all four populations studied. To investigate the possible presence of multiple gene copies, a partial fragment of *Ace-1* was PCR amplified from 12 individuals from Kampala. After cloning (S1 Methods), between six and eight colonies from each individual were sequenced in order to detect the presence of >2 alleles, indicative of gene duplication [15].

Sequences from each individual were aligned in CodonCode Aligner software version 4.2.2 with ClustalW [38] and visualized using Jalview [39]. MEGA 5.1 [40] was used to analyse haplotype variability by calculating the number of polymorphic sites and nucleotide diversity (*π*). Frequencies and relationships between haplotypes were visualised by a minimum spanning network tree generated using the program PopArt available at http://popart.otago.ac.nz.

### Microsatellite genotyping

To date few microsatellite markers have been described for *Cx quinquefasciatus* [41–44], limiting the scope of population genetic studies. Herein, the microsatellite genotyping was conducted using a novel panel of 30 microsatellites loci developed for this study (S2 Methods), which have been combined into five multiplex reactions, thereby enhancing the throughput screening for genetic diversity in *Cx*. *quinquefasciatus*.

The newly designed microsatellite markers were isolated by scanning 180 *Cx*. *quinquefasciatus* supercontigs downloaded from VectorBase [45] with SciRoKo [46]. Individual mosquitoes were typed using five six-plex PCR reactions followed by genotyping using a Beckman-Coulter CEQ8000 capillary electrophoresis system with a 400 size standard kit. Genotypes were sized using the Beckman-Coulter CEQ 2000 DNA analysis system software and manually verified. Microsatellite genotype data were analysed with the program Micro-Checker [47] to detect possible scoring errors (stutter peaks and allele drop-out) and null alleles.

### Microsatellite data analysis

#### Genetic diversity

Following genotyping of 162 mosquitoes at 30 microsatellite loci, four loci were rejected due to presence of null alleles (see Results). Thus, 26 loci were selected for inferences of genetic diversity and population structuring. Allelic frequencies, observed (*H*_O_) and expected (*H*_E_) heterozygosities were calculated using GenAIEX 6.5 [48]. Allelic richness, adjusted to the smallest population sample size, was estimated in FSTAT [49], and Polymorphic Information Content (PIC) calculated using Cervus 3.0 [50]. Genotypic frequencies were tested for deviation from Hardy-Weinberg equilibrium (HWE) for each locus by the exact probability test available in GENEPOP 4.3 [51], followed by a sequential Bonferroni correction.

#### Genetic structure

Genetic differentiation among populations was estimated by pairwise *F*_*ST*_ using Arlequin 3.5.1 [52]. In addition, an AMOVA analysis was also carried out in Arlequin to estimate the level of differentiation among populations from different clusters based on *F*_*ST*_ values. The pattern of migration under an isolation-by-distance model was tested with a Mantel’s test using distance linearized *F*_*ST*_ (*F*_*ST*_/(1-*F*_*ST*_)) and geographic distance [53]. Geographic distance corresponding to the great-circle distance, which corresponds to the shortest distance over the earth’s surface, was calculated between pairs of geographic coordinates (using http://www.movable-type.co.uk/scripts/latlong.html).

A Bayesian analysis to infer the population structure without prior information of the geographic distribution of samples was carried out using STRUCTURE 2.3 [54]. To identify the optimal number of clusters (*K*) in these populations twenty independent runs were conducted for each *K* value (ranging from *K* = 1 to *K* = 7) with 10,000 interactions and 100,000 replications. The most likely *K* value was calculated for each run by the log likelihood (LnP(D)) method and results compiled using CLUMPP [55]. Population structure was also evaluated by Discriminant Analysis of Principal Components (DAPC) using the adegenet R package [56] [57]. To identify the optimal number of clusters for the DAPC clustering, k-means values were sequentially tested, and then k values compared using Bayesian Information Criterion (BIC), with the lowest value of BIC used as the likely number of clusters

#### Gene flow

To infer levels of gene flow among populations we utilized a Bayesian approach implemented in BAYESSASS 3.0 [58]. Initially, five independent runs were performed applying the parameters 10^7^ MCMC, 10^6^ burn-in, chain sampling every 2,000 generations, and all mixing parameters set as default (0.10), with the exception of both allele frequencies (DA) and inbreeding coefficient (DF) runs set at 0.30 as suggested by the author. Parallel runs differed only in initial seed value. Mixing and convergence of MCMCs were visually assessed using TRACER 1.6. Among the five independent runs, we chose the one with lowest Bayesian deviation and constant convergence to assess migration patterns.

#### Detection of loci under selection

The 26 microsatellite loci studied were tested for selection based on the *F*_*ST*_ outlier approach. This method evaluates the relationship between *F*_*ST*_ and *H*_*E*_ (expected heterozygosity) under the assumption of neutrality. The test was performed under an island model and infinite allele model as implemented in LOSITAN [59]. Analyses were performed applying the “Neutral mean *F*_*ST*_” to allow the program to run once to determine a first candidate subset of selected loci in order to remove them from the computation of the neutral *F*_*ST*_, and then the option “Force mean *F*_*ST*_” was also used to get a simulated mean *F*_*ST*_ close to the observed one found in the real dataset. All other options or settings were left as default and 50,000 simulations were run.

To investigate if differing selection strength is detectable between geographic locations the *F*_*ST*_ outlier method was run, with all populations considered separately and with Central Ugandan populations (largely urban) combined. Thus, four simulations were performed as described above for: (a) all populations, (b) populations defined here as Central Uganda (Jinja and Kampala) vs Tororo, (c) Central Uganda vs Kanungu and (d) Kanungu vs Tororo. Where signatures of genomic selection in the vicinity of likely candidate microsatellite selected markers were detected, we examined the number of genes present in the supercontig encompassing the microsatellite markers and their GO terms using the BioMart tools available at VectorBase (www.vectorbase.org).

## Results

### Frequency of target-site resistant alleles

Genotyping of two target-site mutations, *Ace1*-*119S* and *Vgsc*-*1014F*, was conducted utilizing TaqMan allelic discrimination and pyrosequencing assays, respectively (S1 Results). The *Ace-1* resistant allele (AGC) was observed in all populations with a frequency ranging from 12–32% (Fig 1A). Despite the relatively high frequency of the 119S allele, we observed a complete absence of homozygous resistant mosquitoes (S1 Fig). The frequency of heterozygotes was almost three times higher in Kampala and Jinja compared to the other two populations (Fig 1B). Among the four populations a moderate *H*e (0.323 ± 0.065) was observed with a marked excess of heterozygotes (*F*_IS_ = -0.362), whilst within populations, genotype frequencies were not significantly different from Hardy-Weinberg expectation for both the Kanungu and Tororo populations (Table 1). Additionally, no significant difference in resistance allele frequencies was detected between Jinja and Kampala (*P* = 1.0) and between Kanungu and Tororo (*P* = 1.0).

**Table 1:** Ugandan *Culex quinquefasciatus* populations and genotypes of the target-site mutations *Ace1*-G119S and *Vgsc*-L1014F.

	*Ace1-G119S*								*Vgsc-L1014F*										
			Genotype								Genotype								
Population	N	*f* 119S	GG	GS	SS	*H*e	*F*_*IS*_	HW^a^	N	*f 1*014F	LL	F^C^ L	F^C^ F^C^	F^T^L	F^C^F^T^	F^T^ F^T^	*H*e	*F*_*IS*_	HW^a^
Jinja	40	0.32	14	26	0	0.439	-0.481	0.002**	34	0.62	5	13	7	3	5	1	0.611	-0.011	0.945^NS^
Kampala	46	0.32	17	29	0	0.432	-0.46	0.002**	46	0.62	6	16	7	7	8	2	0.649	-0.05	0.954^NS^
Kanungu	45	0.12	34	11	0	0.215	-0.139	0.350^NS^	42	1	0	0	6	0	26	10	0.495	-0.249	0.106^NS^
Tororo	43	0.12	33	10	0	0.206	-0.132	0.388^NS^	43	0.97	1	1	19	0	20	2	0.45	-0.085	0.000***

N is the number of mosquitoes analysed; *H*e is the expected heterozygosity and *F*_*IS*_ is the inbreeding coefficient. HW^a^, *P*-value of *χ*^*2*^ tests for Hardy-Weinberg equilibrium.

*f* 119S and *f* 1014F, correspond to the allele frequency of the *Ace1*-*119S* and *Vgsc*-*1014F* resistant alleles.

GG, GS and SS correspond to homozygous and heterozygous genotypes for the *Ace1*-*G119S* locus.

F^C^; codon TTC, F^T^; codon TTT, L; codon TTA are distinct alleles at the *Vgsc*-*L1014F* locus.

^NS^, not significant;

**P < 0.01;

***P < 0.001.

The *Vgsc*-*1014F* locus in *Cx*. *quinquefasciatus* has two alternative non-synonymous substitutions (TTT and TTC) at the third position of the codon (L1014F) [60]. For the *Vgsc*-*1014F* mutations we detected a high frequency of resistant alleles ranging from 62–100% (Fig 1A). The pyrosequencing genotyping identified 11 individuals harbouring all three *kdr* alleles instead of the two expected for diploid organisms (S3A Fig). Mosquitoes with tri-allelic genotypes were observed in all populations with the exception of Kanungu, with a frequency of 4.0–10.8% (S3B Fig). Excluding individuals with this tri-allelic pattern to allow the use of standard methods to infer departures from Hardy-Weinberg, our analysis detected significant deviation from HWE only in mosquitoes from Tororo (Table 1).

Significant differences in *Vgsc* resistance allele frequencies were detected between all populations with the exception of the Jinja and Kampala comparison (*P* = 0.63). The TTC resistant allele was observed at a high frequency (41–69%) in all populations with the exception of Kanungu, where a higher frequency of the TTT resistant allele (55%) was detected, coupled with an absence of the wild-type allele (Fig 1A). In all populations, we observed a low frequency of susceptible homozygotes (ranging from 0 to 14%), while the majority of resistant alleles were observed in heterozygotes (Fig 1B).

### *Ace-1* haplotype diversity and identification of allelic copy variation

A 535 bp fragment of the *Ace-1* gene was sequenced from 12 individuals (*N* = 57 sequences with between six and eight colonies sequenced from each individual; GenBank accession numbers: KT591708-KT591765) with nine haplotypes detected displaying a total of 10 polymorphic sites (S3 Table) and haplotype frequencies ranging from 0.017–0.397. The resistant allele was detected in four haplotypes: A, E, H and I accounting for 40% of total haplotypes identified. The minimum spanning network tree (S3C Fig) shows that three resistance bearing haplotypes (A, H, I) differ from each other by a single mutational step with Haplotype A the most common. The remaining 119S haplotype (E) is equally distant from both haplotypes A and B differing by three mutational steps, while it differs by only two mutational steps from haplotype D. For wildtype *Ace1*-*119G*, three haplotypes B, C and G were detected with similar frequencies whereas haplotypes D and F were the least frequent. From 12 individuals studied through cloning and sequencing we identified three individuals with more than two distinct *Ace-1* haplotypes, indicative of duplication. These three mosquitoes displayed from three to five haplotypes in the neighbor-joining dendrogram (S3D Fig).

### Microsatellite genotyping

#### Genetic diversity

A total of 186 alleles were detected across the 30 microsatellite loci (S4 Table, S2 Results) with 19 private alleles detected in 17 of the loci. The number of unique alleles in the sample from Kampala was twice that detected in any other population (S2 Fig); however, the presence of these eight private alleles are unlikely to significantly influence the genetic structure analysis since only two alleles have a frequency higher than 1%. The allelic richness and Polymorphic Information Content (PIC) across markers varied from 2.88 (MCQ2) to 11.14 (MCQ21) and from 0.332 (MCQ2) to 0.825 (MCQ26), respectively (S4 Table).

Hardy-Weinberg exact tests indicated that 26 out of 120 instances did not conform to HW equilibrium after multiple-testing correction. However, only two loci (MCQ28 and MCQ33) showed departure from HW equilibrium across all populations (S5 Table). The number of loci that were not in HW equilibrium in each population ranged from 5 to 8 (S2 Table). For all loci with departure from HW equilibrium, significant deviation was associated with a positive inbreeding coefficient (*F*_*IS*_), revealing deviation in the direction of heterozygote deficiency. Analyses performed with Micro-Checker indicated a high frequency of null alleles at loci MCQ28 (0.12) and MCQ33 (0.22), which could likely be responsible for the heterozygote deficiency at these loci. Although loci MCQ1 and MCQ34 were within HW expectation in at least one population, these loci failed to amplify in more than 10% of the samples also suggesting the presence of null alleles. We therefore excluded four markers (MCQ1, MCQ28, MCQ33 and MCQ34) of the 30 tested. The microsatellite exclusion level in this study (four of 30 microsatellites or 13%) is consistent with previous studies which have examined genotypes in family lines as an additional verification of utility (e.g. [43, 61]).

After removal of these four markers from the analysis due to the likely presence of null alleles, levels of genetic diversity, allelic diversity, expected heterozygosity (*H*_E_) and allelic richness within populations were similar across populations (S2 Fig, S5 Table). The mean number of alleles across all loci varied from 4.96 in samples from Kanungu to 5.42 in samples from Kampala, with the highest allelic richness (*R*s = 5.17) detected in Kampala. Nevertheless, no significant difference in the index of genetic diversity was observed among the populations (S2 Fig). Average locus *H*_*E*_ across all samples ranged from 0.597 in Kampala to 0.612 in Kanungu with a median of 0.604 (S2 Table).

#### Population structure

**Fig 2:**
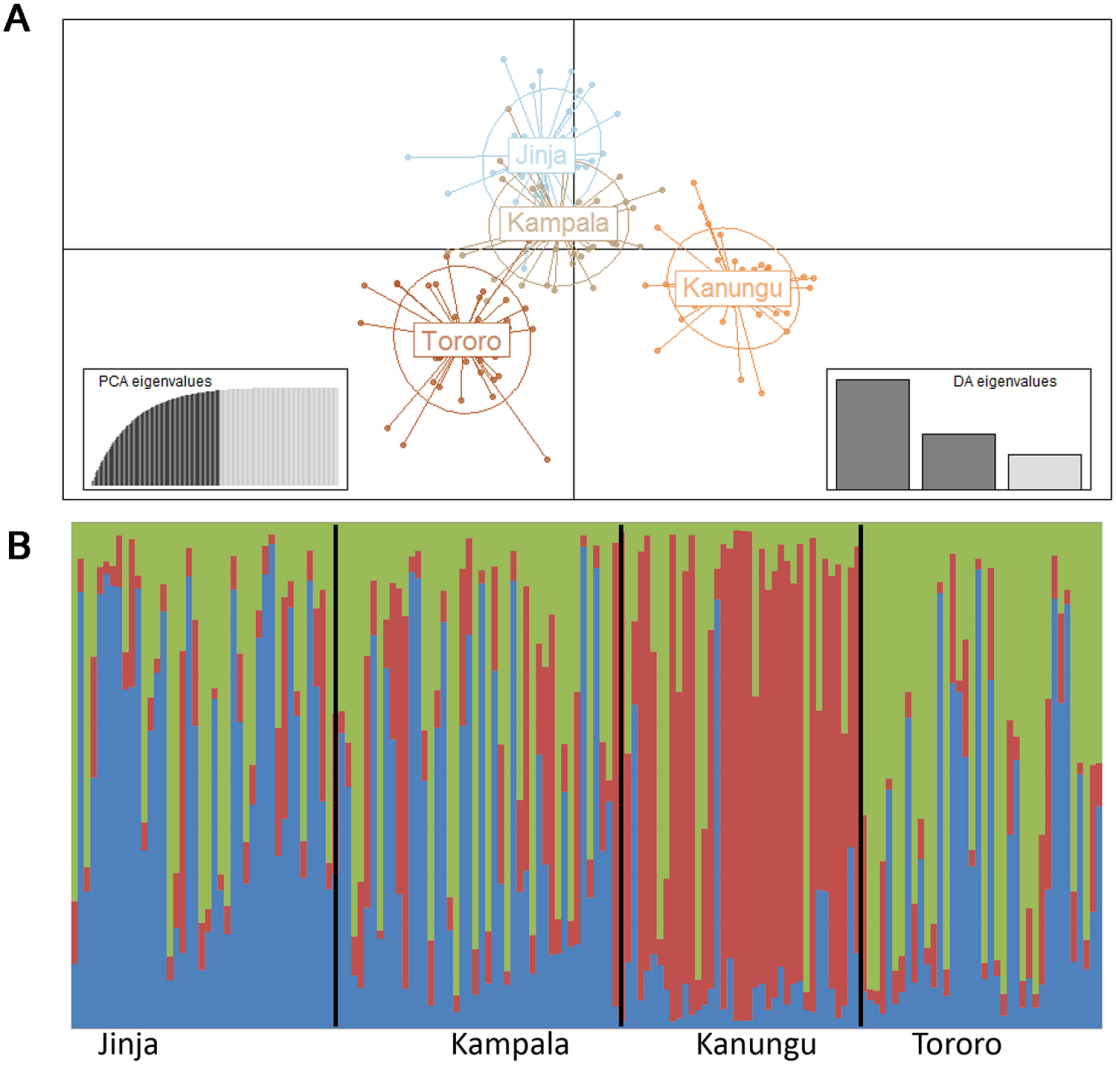
Genetic differentiation estimates among the four *Cx*. *quinquefasciatus* populations based on allele frequencies at 26 microsatellite markers. (A) First and second PCs of the Discriminant Analysis of Principal Components (DAPC) to infer population structure. The graph represents the individuals as dots and the groups as coloured ellipses, which model 95% of the corresponding variability plotted. Populations (ellipses) are plotted within the orthogonal space defined by the first two PCA eigenvalues (inserts). (B) Bayesian analysis of population structure performed using STRUCTURE. Diagrammatic representation of population clusters for the most likely *K* (*K* = 3) with each coloured segment corresponding to the proportion of individuals assigned to a hypothetical population or cluster. Each vertical bar represents an individual with the height of the column segments showing the probability of assignment of belonging to one of the three clusters. Dark lines correspond to population assignment based on geographic collection-site.

Pairwise *F*_*ST*_ estimates were used to measure the genetic differentiation among the four Ugandan populations. The overall *F*_*ST*_ value was relatively low but statistically significant (*F*_*ST*_ = 0.019, *P* = 0.001), while the pairwise *F*_*ST*_ values between populations ranged from 0.0071 to 0.0293 (S6 Table), with all figures significant at *P* < 0.05. The DAPC and Structure analysis (S4 Fig) also indicated population structure, which grouped the four populations within three clusters *K* = 3, with individuals from Jinja and Kampala belonging to the same cluster and this being distinct from the other two samples (Fig 2A and 2B). AMOVA analysis carried out using the four populations showed that the majority of variation exists within populations (83%), while the variation among individuals and among populations are 15 and 2% respectively (S7 Table).

#### Gene flow

Levels of migration between the studied populations were measured under Bayesian inference and this substantiated the existence of contemporary migration in the studied populations. The proportion of individuals originating within each population varied from 67.5% to 93.5%, with the highest admixture in Kampala and Kanungu which have the lowest proportions of individuals originating within the respective population (Table 2). Non-symmetrical rates of gene flow between populations, ranging from 0.71% - 14.5% of individuals originating from a different population, are also shown in Table 2. Posterior probability of migration among populations was inferred using the run with the lowest estimate of Bayesian deviance (among five independent runs, S5A–S5E Fig), with mixing parameter adjusted as migration rates; 0.1, allele frequency; 0.3 and inbreeding coefficients; 0.30.

**Table 2:** Matrix of inferred gene flow between Ugandan *Cx*. *quinquefasciatus* populations.

**Gene Flow**	**Jinja**	**Kampala**	**Kanungu**	**Tororo**
**Jinja**	0.9349(0.0334)	0.0080(0.0079)	0.0085(0.0083)	0.0486(0.0322)
**Kampala**	0.2911(0.0201)	0.6745(0.0076)	0.0079(0.0079)	0.0265(0.0175)
**Kanungu**	0.1725(0.0342)	0.0079(0.0076)	0.6750(0.0082)	0.1446(0.0339)
**Tororo**	0.1150(0.0508)	0.0079(0.0077)	0.0092(0.0090)	0.8678(0.0510)

Values in the form mij representing the proportion of individuals in the i^th^ population that originated from the j^th^ population per generation. Values in parentheses are standard deviations of the posterior probability distributions.

**Fig 3:**
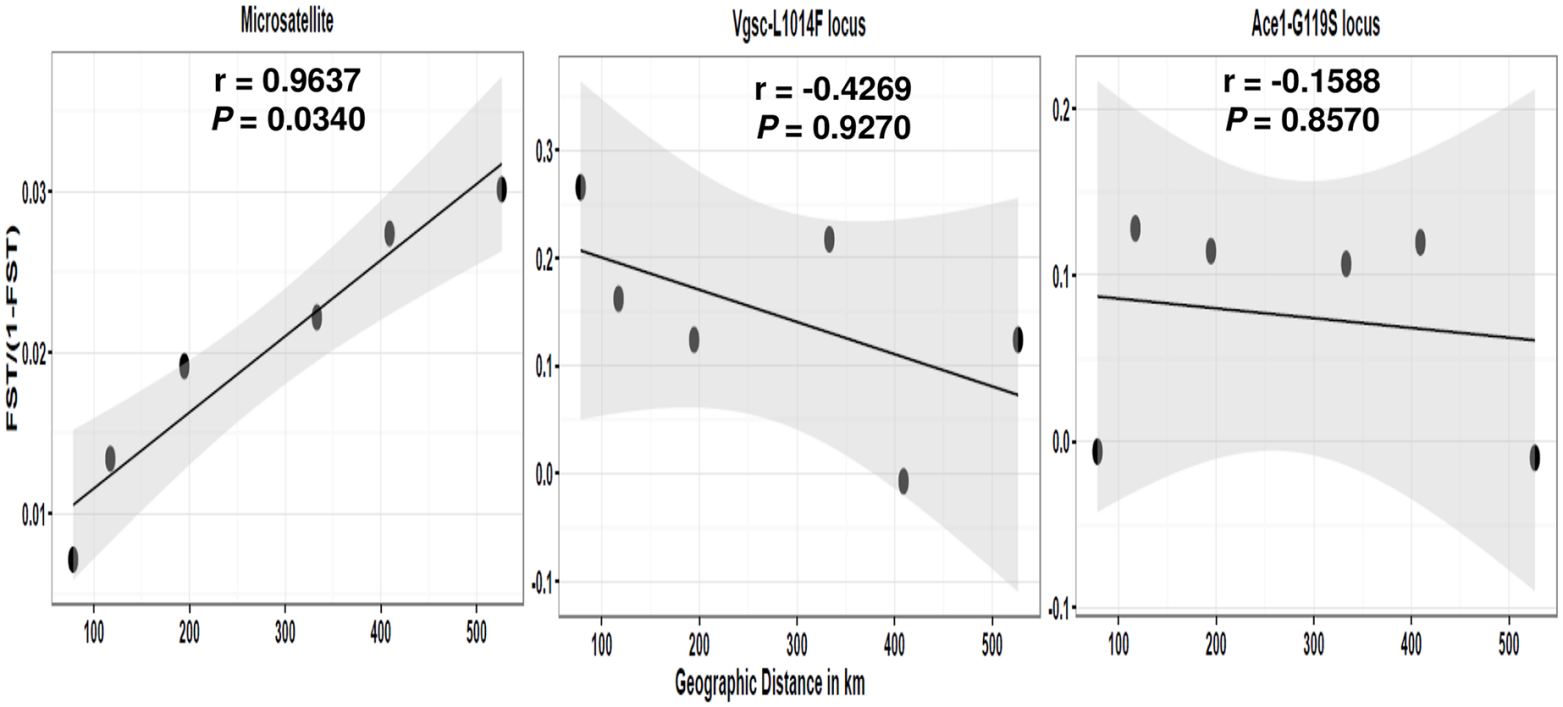
Relationship between geographic distance versus genetic distance (*F*_*ST*_/1- *F*_*ST*_), analysed in parallel for the microsatellite, *Vgsc*-*L1014F* and *Ace1-G119S* loci. Significance was tested using a Mantel test. Grey shading corresponds to the 95% confidence interval. Geographic distance corresponding to the great-circle distance, which corresponds to the shortest distance over the earth’s surface between two points.

Analysis of isolation-by-distance also supports a pattern of migration, with migration between populations positively correlated with the geographic distance (R^2^ = 0.963, *P* = 0.047) based on microsatellite markers (Fig 3). In contrast to microsatellite markers, for both insecticide resistance loci *Vgsc-1014F* and *Ace1*-*119S*, no significant association was detected between the frequency of resistant alleles and a sample’s geographical location (Fig 3).

#### Loci under selection

**Fig 4:**
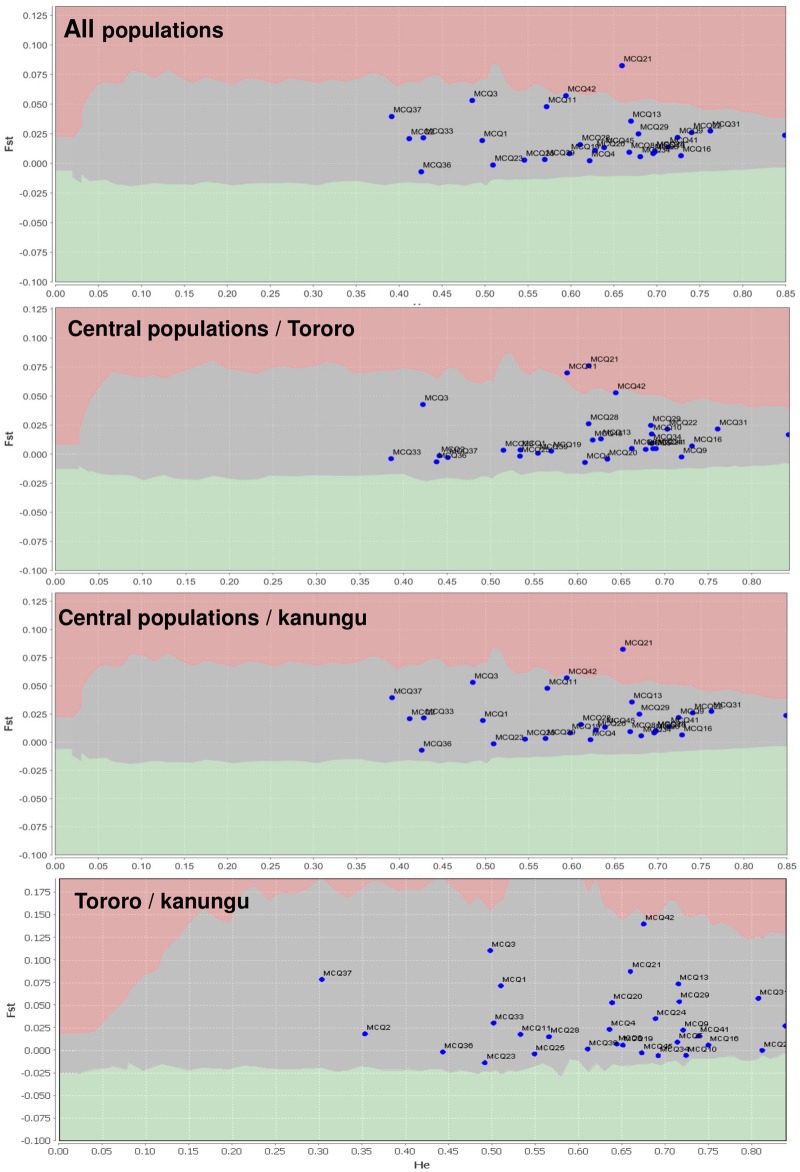
*F*_*ST*_ outlier approach for the 26 studied microsatellite loci versus a model of neutral evolution in Ugandan *Cx*. *quinquefasciatus*. Central populations correspond to a cluster of Jinja and Kampala (based on genetic structure analysis, see Fig 3). Each dot represents a microsatellite marker. The markers located in the grey middle area are assumed to be neutral. The markers located below the neutral area are candidates for being under balancing selection and the markers located above the neutral area are candidates for being under directional selection. *X*-axis: estimated heterozygosity values. *Y*-axis: *F*_*ST*_-values. Analyses were performed assuming an infinite allele model.

The 26 loci were also tested for selection based on the *F*_*ST*_ outlier approach, from which only marker MCQ21 showed strong signs of divergent selection when comparing all populations, or in comparisons of the two distinct sub-sets of populations based on population genetic structuring—Central Uganda (Jinja and Kampala) compared to Tororo and Kanungu. No signal of selection was observed for MCQ21 when comparing only Tororo and Kanungu (Fig 4). Marker MCQ11 showed a signal of divergent selection only when comparing Central Uganda and Tororo (Fig 4). Supercontigs containing these markers (3.186 for MCQ21 and 3.526 for MCQ11) contain 47 and 22 genes respectively in the vicinity of these markers with a genomic window resolution of 500 kb and 347 kb, respectively (S3 and S4 Results). No genes with recognised links to insecticide resistance were observed (e.g. detoxification gene families such as the cytochrome P450 (P450s), esterases and glutathione S-transferases (GSTs))

## Discussion

Resistance alleles at the target sites of insecticides have known deleterious effects [13, 62] and are therefore not expected to persist in the absence of selection. Our data which indicate copy number variation at both the *Vgsc*-*L1014F* and *Ace1*-*G119S* loci and allele frequency differences between populations for both loci suggest differential insecticide selection pressure on *Cx*. *quinquefasciatus* populations across Uganda. The significantly higher frequency of *Vgsc*-*1014F* in comparison to *Ace1*-*119S* for all populations has been reported previously in *Culex* and other vector mosquitoes on the African continent [63–65], and might result from past and ongoing reliance on pyrethroid-based vector control approaches to mitigate the burden of vector-borne diseases. However, it is important to bear in mind that our allele frequency estimates are likely under-reporting true frequencies, particularly for the *Vgsc*, as the duplication observed in Ugandan populations imposes limitations on available methods to infer allele frequency as reported elsewhere [37].

Whilst recognising these limitations, our data show a contrasting pattern of *Ace1-119S* and *Vgsc*-*1014F* allele frequencies across populations, likely indicating heterogeneous selection pressure. For both markers, we were not able to identify a clear spatial geographic spread, but we did detect heterogeneity in allele frequencies of these selected markers when comparing Uganda Central to Eastern and Southwest population. Indeed, the highest frequency of *Vgsc-1014F* was detected outside the Central region, while the highest frequency of *Ace1-119S* was observed with a pattern of geographic distribution opposite to that of *Vgsc-1014F*. Therefore, these figures highlight the challenges for implementation of efficient control intervention in highly fragmented habitats including overlapping of urban and rural patches or due to heterogeneous urbanization structure, which could result in a mosaic of landscapes with sharp shifts of insecticide selection pressure and frequency of insecticide-resistance associated alleles [66–68].

Markedly, although our study demonstrates high frequencies of both target-site mutations (*Vgsc*-*1014F* and *Ace1*-*119S*) in Ugandan *Cx*. *quinquefasciatus* populations no vector control programme targeting this species has been conducted. Therefore, these results support a hypothesis that the evolution of insecticide resistance in these populations is driven by control interventions targeting other vector species or pest insects. Indeed, previous studies have suggested that the evolution of insecticide resistance in *Cx*. *quinquefasciatus* on the African continent might result from intervention approaches targeting sympatric *Anopheles* species [27, 69]. For instance, a study of *Cx*. *quinquefasciatus* from Zambia showed a significant increase of the *Vgsc*-*1014F* mutation and increase of detoxification enzyme activity assays after introduction of ITNs for malaria control [27].

In Uganda in particular, we suggest that variation of *Vgsc*-*1014F* frequency between districts might reflect the combination of approaches applied to reduce *Anopheles* populations, including national distribution of ITN and IRS only in highly endemic and epidemic-prone regions. For example, across Uganda irregular spraying with insecticides such as lambda-cyhalothrin, DDT, alpha cypermethrin and latterly bendiocarb [70] has been reported since 2006 when IRS was re-introduced for vector control [71]. Regional differences in ITN coverage may also contribute to the *Vgsc-1014* allele distribution e.g. the Uganda Bureau of Statistics [72] report that householders that own at least one ITN differ by 44% between the East Central and West Nile regions in 2011.

Markedly, we have also noticed a distinct pattern of ITN usage among UDHS, which differ by 14% between Central-South western and 8.7% Central-Mid-eastern, despite a similar ITN coverage of around 58% for all three regions (S1 Table). Additionally, the contrasting pattern of *Vgsc*-1014 markers among the studied districts might also reflect the drastic shift of ITN distribution, which increased by 26% (Central region), 41.6% (Mid-eastern) and 32.8% (South-western) between 2006 and 2009 [32, 33].

Nevertheless, with our study design it is not possible make a direct association of the UNMCP (Uganda National Malaria Control Program) interventions with the evolution of resistance in *Culex* populations. Indeed, we recognise that pinpointing the particular source underlying the evolution of resistance remains a difficult task. For example, increased resistance in *Cx*. *quinquefasciatus* populations from Ghana and Benin, with high frequencies of *Ace1*-*119S* was attributed to the use of organophosphates and/or carbamate insecticides by farmers for pest control or pollutants in mosquito breeding sites [66, 67]. Heavy use of domestic insecticide was also associated with increased insecticide resistance in *Cx*. *quinquefasciatus* populations from Ghana [73].

Regardless of the strength of selection imposed by diverse insecticide-based approaches such as ITNs, IRS, pesticides and environment pollutants [23], the evolutionary pattern of increased resistance in *Cx*. *quinquefasciatus* from Uganda could also be modulated by variation in the repertoire of adaptive genetic mechanisms, as in the studied population we identified gene duplication for both target-site loci and allelic variation of the *Vgsc* resistant allele. Interestingly, we identify two alternative resistant alleles (TTT and TTC) in the *Vsgc*-*1014* codon observed at high frequency in all populations, contrasting with previous reports on the geographic distribution of *Vgsc-1014F* in *Culex* mosquitoes worldwide [11], which exclusively detect the TTT allele [74–76]. To date, the TTC variant has been detected at high frequency only in *Cx*. *quinquefasciatus* from Sri Lanka, which was previously argued as the most likely geographic origin [60], and was only recently detected at very low frequency in African *Cx*. *quinquefasciatus* from Zanzibar [69]. Although we identified co-occurrence of both resistant variants of *Vgsc* it was not possible to determine if heterozygote TTT/TTC increases a mosquito’s level of resistance, or offsets possible deleterious effects.

We also identify a significant difference in frequency of the *Vgsc*-*1014F* resistant alleles among populations, with the TTC variant being predominant in all populations with the exception of Kanungu. We do not have a conclusive explanation for the difference in frequency of TTT and TTC among Ugandan populations and between Uganda and other African populations. Nevertheless, a few potential mechanisms could explain this. One possible explanation involves a colonization process with the TTC allele introduced to Uganda from another geographical region with a large founder effect followed by genetic drift that shifted the TTT and TTC allele frequency, although a *de novo* origin and dispersion cannot be discounted. Alternatively, it is also possible that the TTC allele arose on the African continent but has not been detected in other populations due to the application of specific *Culex* species *Vgsc*-*1014* genotyping assays, which were mostly designed for detecting the TTT and TTA alleles [77].

Our target-site genotyping of the *Ace1*-*119* and *Vgsc*-*1014* variants also indicated the presence of gene duplication for both genes, suggesting that CNV could be among the mechanisms driving the evolution of insecticide in these populations. Indeed, gene duplication of insecticide-associated genes such as *Ace-1*, esterase estα2^1^ and estβ2^1^, *Rdl* (resistance to dieldrin) and detoxification genes (e.g. GSTs, P450s) have been reported as adaptive resistance mechanisms in diverse arthropod species including *Tetranychus urticae*, *Aphis gossypii* and *A*. *gambiae* [3, 14, 16, 37, 78].

The likely duplication of the *Vgsc* was indicated by the presence of 6.25% of mosquitoes genotyped by pyrosequencing displaying three alleles simultaneously for *Vgsc*-*1014*. For *Ace*-*1*, duplication was evidenced by a haplotype analysis, which identified three out of 12 individuals exhibiting either 3 or 5 distinct haplotypes simultaneously. Whilst contamination or DNA from spermatheca could be mistaken for duplications we have shown that the duplication of *Vgsc* is present in males [79] and we see no triallelic genotypes in the microsatellite data. This duplication could explain the large variation observed within resistant and susceptible alleles as demonstrated by the minimum spanning Network (S3C Fig), which contrasted with an expected low haplotype diversity due to a selective sweep effect on a locus under selection.

The absence of *Ace*-*119S* homozygotes in all four populations, was not surprising as the same pattern has been reported previously in both *Culex* and *Anopheles* populations [16, 20, 80]. In both species, this pattern has been linked to the deleterious effects of the homozygous resistant genotype due to changes in acetylcholinesterase kinetic properties and mutation fitness cost in an insecticide-free or reduced insecticide exposure environment. Alternatively, the absence of resistant homozygotes might also arise from duplication of the *Ace-1* gene involving resistant and susceptible alleles, creating a permanent heterozygosis to partially normalise AChE enzyme activity levels [37, 81]. In contrast to the proposed compensatory adaptive mechanism of the *Ace-1* duplication to overcome deleterious effects and fitness disadvantage of the resistant *Ace1*-*119S* [19, 20], the likely adaptive role played by the recently reported *Vgsc* duplication in *Cx*. *quinquefasciatus* from the USA (Xu et al. 2011) and in *A*. *aegypti* from Brazil [22] is currently unknown.

Our study also detected a contrasting pattern between microsatellite and insecticide selected markers. For both markers linked to resistance, it was not possible to identify a clear spatial geographic pattern, but we did detect heterogeneity in allelic frequencies when comparing Ugandan Central (largely urban) to Eastern and Southwest populations (largely rural). By contrast, no significant difference in genetic diversity indices was observed at neutral markers among the populations, although these data did indicate a structured pattern. Although the low levels of genetic variation of microsatellites across Ugandan populations from distinct geographic locations have also been reported in *Cx*. *pipiens* from California, USA [82], heterogeneity in diversity levels across Uganda was expected since vector control interventions have been applied annually, and might be expected to result in population bottlenecks. Such reductions of genetic diversity between populations under different strengths of selection pressure have been reported in *Cx*. *quinquefasciatus* from Recife, Brazil after vector control intervention with *Bacillus sphaericus*, a bio-larvicide [83]. As indicated by these authors, discrepancies between neutral and selected markers might be linked to differences in the marker’s genetic properties that modulate changes in allele frequencies, such as drastic bottleneck effects and differences in intensity of local selection with frequent gene flow [84, 85].

The microsatellite data also indicated significant genetic structure among populations with the three clusters identified by the STRUCTURE and DAPC analyses corresponding to Eastern, Central and Southwest Uganda regions. The level of population structure among our study sites was consistent with findings from similar studies in *Culex* mosquitoes using molecular markers as diverse as ISSR (Inter-Simple Sequence Repeat) and microsatellites, which reported that population structure reflects geoclimatic, geographic distance and environmental variables [86–88]. Additionally, based on microsatellite markers an intense migration among Ugandan populations with significant patterns of isolation by distance (IBD) was detected, which could prevent loss of genetic diversity and consequently could explain the homogenous microsatellite genetic diversity observed across the studied populations.

The distinct pattern of IBD observed for microsatellite markers and selected markers, also indicated that factors other than geographic distance, such as environmental heterogeneity as well as serial sequential founder effects, may also influence the pattern of spatial autocorrelation of resistance-associated markers [89]. For example, in *Aedes rusticus*, comparisons among areas treated and not-treated with *Bacillus thuringiensis israelensis* (*Bti*) show more intense IBD among treated than among non-treated sites [90]. These authors argue that the difference in observed IBD instead of spatial distribution could result from differences in population size after insecticide application or lower migration capability of selected mosquitoes imposed by a fitness cost of the resistance mechanism.

Therefore, despite the intense gene flow identified in the studied populations, it is possible that local differences in strength of insecticide selection might have created heterogeneous “insecticide adaptive islands” across the geographic regions. Analysis of microsatellite locus neutrality indicated that all markers indeed adhered to a neutral model with the exception of the MCQ21 across all populations and MCQ11 when comparing only Ugandan Central to Eastern populations. For both markers, further analysis of the genomic location of markers does not indicate likely linkage with known insecticide associated genes such as *Ace*-*1*, *Vgsc* or genes encoding detoxification enzymes (e.g. esterase, GSTs and P450s). However, this analysis was limited by the incomplete *Cx*. *quinquefasciatus* genome assembly, which restricted the genomic window capable of being applied in the analysis. Due to the contrasting patterns of neutrality for marker MCQ21, we speculate that this marker could be hitchhiking with an insecticide-associated gene nearby this marker, with a genomic location beyond the supercontig resolution.

In conclusion, our study shows that the contemporary pattern of local selection is driven by the evolution of insecticide resistance in Ugandan *Cx*. *quinquefasciatus* occurring despite the absence of vector control intervention targeting this species. This study demonstrates that although it is challenging to pinpoint the direct source of insecticide exposure in a background of selection pressure, studies of the evolution of insecticide in non-target species can provide important information about the nature of the insecticidal challenges to which vectors are exposed.

## Supporting information

S1 Methods Allelic discrimination assays of insecticide target-site mutations in *Culex quinquefasciatus*. (PDF)

S2 Methods Development of microsatellite multiplex panels for population genetic analysis in *Culex quinquefasciatus* mosquitoes. (PDF)

S1 Results *Cx*. *quinquefasciatus* Ugandan *Ace1*-119 and *Vgsc*-1014 genotyping. (XLSX)

S2 Results *Cx*. *quinquefasciatus* Ugandan microsatellite genotyping data. (XLSX)

S3 Results Genomic information for supercontig 3.186. (XLS)

S4 Results Genomic information for supercontig 3.526. (XLS)

S1 Fig *Ace*-*1* target-site mutation (G119S) genotyping in *Cx*. *quinquefasciatus*. (PDF)

S2 Fig Genetic diversity estimates across Ugandan *Cx*. *quinquefasciatus* populations based on 26 microsatellite markers. (PDF)

S3 Fig Detection of copy number variation in the *Vgsc* and *Ace-1* gene in *Cx*. *quinquefasciatus*. (PDF)

S4 Fig Clustering analysis in field-collected Ugandan *Cx*. *quinquefasciatus* using Bayesian assignment implemented in the STRUCTURE software. (PDF)

S5 Fig BAYESASS 3.0 trace-plot and analysis parameters from five parallel runs to detect the lowest Bayesian deviance and examine convergence. (PDF)

S1 Table Location of field-caught mosquitoes, demographic index of collection sites and main features of vector control intervention in Uganda 2011. (PDF)

S2 Table Sample sizes and indices of genetic diversity at 26 microsatellite loci for four Ugandan *Cx*. *quinquefasciatus* populations. (PDF)

S3 Table *Ace-1* haplotype diversity based on sequences obtained from *Cx*. *quinquefasciatus* mosquitoes sampled in Uganda. Polymorphic sites identified in a 535 bp partial fragment (intron 2 and exon 3) of *Ace-1*.
(PDF)

S4 Table Microsatellite locus characterization across all *Cx*. *quinquefasciatus* populations. (PDF)

S5 Table Summary of microsatellite variation in different Ugandan populations of *Cx*. *quinquefasciatus*. (PDF)

S6 Table Pairwise *F*_*ST*_ estimates (Weir & Cockerham 1984) across Ugandan *Culex quinquefasciatus* populations. (PDF)

S7 Table Analysis of molecular variance (AMOVA) for four *Culex quinquefasciatus* populations from Uganda, using 26 microsatellite markers. (PDF)

## References

[1] Nauen R. Insecticide resistance in disease vectors of public health importance. Pest Management Science. 2007;63(7):628–33. doi: 10.1002/ps.1406 17533649 10.1002/ps.1406

[2] Karunamoorthi K, Sabesan S. Insecticide resistance in insect vectors of disease with special reference to mosquitoes: a potential threat to global public health. Health Scope. 2013;2(1):4–18.

[3] Li X, Schuler MA, Berenbaum MR. Molecular mechanisms of metabolic resistance to synthetic and natural xenobiotics. Annual Review of Entomology. 2007;52:231–53. doi: 10.1146/annurev.ento.51.110104.151104 16925478 10.1146/annurev.ento.51.110104.151104

[4] Liu N. Insecticide resistance in mosquitoes: impact, mechanisms, and research directions. Annual Review of Entomology. 2015;60:537–59. doi: 10.1146/annurev-ento-010814-020828 25564745 10.1146/annurev-ento-010814-020828

[5] Soderlund DM. Pyrethroids, knockdown resistance and sodium channels. Pest Management Science. 2008;64(6):610–6. doi: 10.1002/ps.1574 18383430 10.1002/ps.1574

[6] Davies TGE, Field LM, Usherwood PNR, Williamson MS. DDT, pyrethrins, pyrethroids and insect sodium channels. IUBMB Life. 2007;59(3):151–62. doi: 10.1080/15216540701352042 17487686 10.1080/15216540701352042

[7] Donnelly MJ, Corbel V, Weetman D, Wilding CS, Williamson MS, Black WC IV. Does *kdr* genotype predict insecticide-resistance phenotype in mosquitoes? Trends in Parasitology. 2009;25(5):213–9. doi: 10.1016/j.pt.2009.02.007 19369117 10.1016/j.pt.2009.02.007

[8] Casida JE, Durkin KA. Neuroactive insecticides: targets, selectivity, resistance, and secondary effects. Annual Review of Entomology. 2013;58:99–117. doi: 10.1146/annurev-ento-120811-153645 23317040 10.1146/annurev-ento-120811-153645

[9] Zhao Q, Zhu Z, Kasahara M, Morishita S, Zhang Z. Segmental duplications in the silkworm genome. BMC Genomics. 2013;14 doi: 10.1186/1471-2164-14-521 23901934 10.1186/1471-2164-14-521 PMC3735471

[10] Santolamazza F, Calzetta M, Etang J, Barrese E, Dia I, Caccone A, et al Distribution of knock-down resistance mutations in *Anopheles gambiae* molecular forms in west and west-central Africa. Malaria Journal. 2008;7 doi: 10.1186/1475-2875-7-74 18445265 10.1186/1475-2875-7-74 PMC2405802

[11] Scott JG, Yoshimizu MH, Kasai S. Pyrethroid resistance in *Culex pipiens* mosquitoes. Pesticide Biochemistry and Physiology. 2015;120:68–76. doi: 10.1016/j.pestbp.2014.12.018 25987223 10.1016/j.pestbp.2014.12.018

[12] Rivero A, Vezilier J, Weill M, Read AF, Gandon S. Insecticide control of vector-borne diseases: when is insecticide resistance a problem? PLoS Pathogens. 2010;6(8):e1001000 doi: 10.1371/journal.ppat.1001000 20700451 10.1371/journal.ppat.1001000 PMC2916878

[13] Djogbénou L, Noel V, Agnew P. Costs of insensitive acetylcholinesterase insecticide resistance for the malaria vector *Anopheles gambiae* homozygous for the G119S mutation. Malaria Journal. 2010;9(1):12 doi: 10.1186/1475-2875-9-12 20070891 10.1186/1475-2875-9-12 PMC2816975

[14] Shang Q, Pan Y, Fang K, Xi J, Wong A, Brennan JA, et al Extensive *Ace2* duplication and multiple mutations on *Ace1* and *Ace2* are related with high level of organophosphates resistance in *Aphis gossypii*. Environmental Toxicology. 2014;29(5):526–33. doi: 10.1002/tox.21778 22489048 10.1002/tox.21778

[15] Labbé P, Berthomieu A, Berticat C, Alout H, Raymond M, Lenormand T, et al Independent duplications of the acetylcholinesterase gene conferring insecticide resistance in the mosquito *Culex pipiens*. Molecular Biology and Evolution. 2007;24(4):1056–67. doi: 10.1093/molbev/msm025 17283366 10.1093/molbev/msm025

[16] Djogbénou L, Chandre F, Berthomieu A, Dabire R, Koffi A, Alout H, et al Evidence of introgression of the *Ace*-1(R) mutation and of the *Ace*-1 duplication in West African *Anopheles gambiae s*.*s*. PLoS One. 2008;3(5). doi: 10.1371/journal.pone.0002172 18478097 10.1371/journal.pone.0002172 PMC2377098

[17] Carvalho R, Yang Y, Field LM, Gorman K, Moores G, Williamson MS, et al Chlorpyrifos resistance is associated with mutation and amplification of the acetylcholinesterase-1 gene in the tomato red spider mite, *Tetranychus evansi*. Pesticide Biochemistry and Physiology. 2012;104(2):143–9.

[18] Alout H, Labbé P, Berthomieu A, Pasteur N, Weill M. Multiple duplications of the rare *Ace-1* mutation F290V in *Culex pipiens* natural populations. Insect Biochemistry and Molecular Biology. 2009;39(12):884–91. doi: 10.1016/j.ibmb.2009.10.005 19874892 10.1016/j.ibmb.2009.10.005

[19] Lee SH, Kim YH, Kwon DH, Cha DJ, Kim JH. Mutation and duplication of arthropod acetylcholinesterase: implications for pesticide resistance and tolerance. Pesticide Biochemistry and Physiology. 2015;120:118–24. doi: 10.1016/j.pestbp.2014.11.004 25987229 10.1016/j.pestbp.2014.11.004

[20] Labbé P, Milesi P, Yébakima A, Pasteur N, Weill M, Lenormand T. Gene-dosage effects on fitness in recent adaptive duplications: *Ace-1* in the mosquito *Culex pipiens*. Evolution. 2014;68:2092–101. doi: 10.1111/evo.12372 24494966 10.1111/evo.12372

[21] Xu Q, Tian L, Zhang L, Liu N. Sodium channel genes and their differential genotypes at the L-to-F *Kdr* locus in the mosquito *Culex quinquefasciatus*. Biochemical and Biophysical Research Communications. 2011;407(4):645–9. doi: 10.1016/j.bbrc.2011.03.060 21419752 10.1016/j.bbrc.2011.03.060 PMC3134871

[22] Martins AJ, Brito LP, Linss JGB, Rivas GBdS, Machado R, Bruno RV, et al Evidence for gene duplication in the voltage-gated sodium channel gene of *Aedes aegypti*. Evolution, Medicine and Public Health. 2013;2013(1):148–60. doi: 10.1093/emph/eot012 . 24481195 10.1093/emph/eot012 PMC3868448

[23] Nkya TE, Akhouayri I, Kisinza W, David J-P. Impact of environment on mosquito response to pyrethroid insecticides: facts, evidences and prospects. Insect Biochemistry and Molecular Biology. 2013;43(4):407–16. doi: 10.1016/j.ibmb.2012.10.006 23123179 10.1016/j.ibmb.2012.10.006

[24] Bockarie MJ, Pedersen EM, White GB, Michael E. Role of vector control in the Global Program to Eliminate Lymphatic Filariasis. Annual Review of Entomology. 2009;54:469–87. doi: 10.1146/annurev.ento.54.110807.090626 18798707 10.1146/annurev.ento.54.110807.090626

[25] Kolaczinski JH, Kabatereine NB, Onapa AW, Ndyomugyenyi R, Kakembo ASL, Brooker S. Neglected tropical diseases in Uganda: the prospect and challenge of integrated control. Trends in Parasitology. 2007;23(10):485–93. doi: 10.1016/j.pt.2007.08.007 17826335 10.1016/j.pt.2007.08.007 PMC2682772

[26] Onapa AW, Simonsen PE, Baehr I, Pedersen EM. Rapid assessment of the geographical distribution of lymphatic filariasis in Uganda, by screening of schoolchildren for circulating filarial antigens. Annals of Tropical Medicine and Parasitology. 2005;99(2):141–53. doi: 10.1179/136485905X19829 15814033 10.1179/136485905X19829

[27] Norris LC, Norris DE. Insecticide resistance in *Culex quinquefasciatus* mosquitoes after the introduction of insecticide-treated bed nets in Macha, Zambia. Journal of Vector Ecology. 2011;36(2):411–20. doi: 10.1111/j.1948-7134.2011.00182.x 22129413 10.1111/j.1948-7134.2011.00182.x PMC4119017

[28] Hoffmann AA, Willi Y. Detecting genetic responses to environmental change. Nature Reviews Genetics. 2008;9(6):421–32. doi: 10.1038/nrg2339 18463665 10.1038/nrg2339

[29] Selkoe KA, Toonen RJ. Microsatellites for ecologists: a practical guide to using and evaluating microsatellite markers. Ecology Letters. 2006;9(5):615–29. doi: 10.1111/j.1461-0248.2006.00889.x 16643306 10.1111/j.1461-0248.2006.00889.x

[30] Uganda Bureau of Statistics. Uganda Demographic and Health Survey 2011. Kampala, Uganda: Uganda Bureau of Statistics, Maryland: ICF International Inc.2012.

[31] Uganda Bureau of Statistics. Republic of Uganda National Population and Housing Census 2014: Provisional Results. Kampala, Uganda.2014.

[32] Uganda Bureau of Statistics (UBOS) and ICF Macro. Uganda Malaria Indicator Survey 2009. Calverton, Maryland, USA: UBOS and ICF Macro, 2010.

[33] Uganda Bureau of Statistics (UBOS) and Macro International Inc. Uganda Demographic and Health Survey 2006. Calverton, Maryland, USA: UBOS and Macro International Inc, 2007.

[34] Abeku TA, Helinski MEH, Kirby MJ, Ssekitooleko J, Bass C, Kyomuhangi I, et al Insecticide resistance patterns in Uganda and the effect of indoor residual spraying with bendiocarb on *kdr* L1014S frequencies in *Anopheles gambiae s*.*s*. Malaria Journal. 2017;16(1):156 doi: 10.1186/s12936-017-1799-7 28427415 10.1186/s12936-017-1799-7 PMC5397803

[35] Bukirwa H, Yau V, Kigozi R, Filler S, Quick L, Lugemwa M, et al Assessing the impact of indoor residual spraying on malaria morbidity using a sentinel site surveillance system in Western Uganda. The American Journal of Tropical Medicine and Hygiene. 2009;81(4):611–4. doi: 10.4269/ajtmh.2009.09-0126 19815875 10.4269/ajtmh.2009.09-0126

[36] Smith JL, Fonseca DM. Rapid assays for identification of members of the *Culex* (*Culex*) pipiens complex, their hybrids, and other sibling species (Diptera: Culicidae). American Journal of Tropical Medicine and Hygiene. 2004;70(4):339–45. 15100444

[37] Kwon DH, Clark JM, Lee SH. Extensive gene duplication of acetylcholinesterase associated with organophosphate resistance in the two-spotted spider mite. Insect Molecular Biology. 2010;19(2):195–204. doi: 10.1111/j.1365-2583.2009.00958.x 20002213 10.1111/j.1365-2583.2009.00958.x

[38] Larkin MA, Blackshields G, Brown NP, Chenna R, McGettigan PA, McWilliam H, et al Clustal W and Clustal X version 2.0. Bioinformatics. 2007;23(21):2947–8. doi: 10.1093/bioinformatics/btm404 17846036 10.1093/bioinformatics/btm404

[39] Waterhouse AM, Procter JB, Martin DMA, Clamp M, Barton GJ. Jalview Version 2—a multiple sequence alignment editor and analysis workbench. Bioinformatics. 2009;25(9):1189–91. doi: 10.1093/bioinformatics/btp033 19151095 10.1093/bioinformatics/btp033 PMC2672624

[40] Tamura K, Peterson D, Peterson N, Stecher G, Nei M, Kumar S. MEGA5: molecular evolutionary genetics analysis using maximum likelihood, evolutionary distance, and maximum parsimony methods. Molecular Biology and Evolution. 2011;28(10):2731–9. doi: 10.1093/molbev/msr121 21546353 10.1093/molbev/msr121 PMC3203626

[41] Edillo F, Kiszewski A, Manjourides J, Pagano M, Hutchinson M, Kyle A, et al Effects of latitude and longitude on the population structure of *Culex pipiens* s.l., vectors of West Nile Virus in North America. American Journal of Tropical Medicine and Hygiene. 2009;81(5):842–8. doi: 10.4269/ajtmh.2009.08-0605 19861620 10.4269/ajtmh.2009.08-0605 PMC2842826

[42] Fonseca DM, Atkinson CT, Fleischer RC. Microsatellite primers for *Culex pipiens quinquefasciatus*, the vector of avian malaria in Hawaii. Molecular Ecology. 1998;7(11):1617–9. 9819914

[43] Hickner PV, deBruyn B, Lovin DD, Mori A, Behura SK, Pinger R, et al Genome-based microsatellite development in the *Culex pipiens* complex and comparative microsatellite frequency with *Aedes aegypti* and *Anopheles gambiae*. PLoS One. 2010;5(9). doi: 10.1371/journal.pone.0013062 20927334 10.1371/journal.pone.0013062 PMC2948009

[44] Smith JL, Keyghobadi N, Matrone MA, Escher RL, Fonseca DM. Cross-species comparison of microsatellite loci in the *Culex pipiens* complex and beyond. Molecular Ecology Notes. 2005;5(3):697–700. doi: 10.1111/j.1471-8286.2005.01034.x

[45] Megy K, Emrich SJ, Lawson D, Campbell D, Dialynas E, Hughes DST, et al VectorBase: improvements to a bioinformatics resource for invertebrate vector genomics. Nucleic Acids Research. 2012;40(D1):D729–D34. doi: 10.1093/nar/gkr1089 22135296 10.1093/nar/gkr1089 PMC3245112

[46] Kofler R, Schloetterer C, Lelley T. SciRoKo: a new tool for whole genome microsatellite search and investigation. Bioinformatics. 2007;23(13):1683–5. doi: 10.1093/bioinformatics/btm157 17463017 10.1093/bioinformatics/btm157

[47] Van Oosterhout C, Hutchinson WF, Wills DPM, Shipley PI. MICRO-CHECKER: software for identifying and correcting genotyping errors in microsatellite data. Mol Ecol Notes. 2004;4(3):535–8.

[48] Peakall R, Smouse PE. GenAlEx 6.5: genetic analysis in Excel. Population genetic software for teaching and research-an update. Bioinformatics. 2012;28(19):2537–9. doi: 10.1093/bioinformatics/bts460 22820204 10.1093/bioinformatics/bts460 PMC3463245

[49] Goudet J. FSTAT, a program to estimate and test gene diversities and fixation indices (version 2.9. 3). 2001.

[50] Kalinowski ST, Taper ML, Marshall TC. Revising how the computer program CERVUS accommodates genotyping error increases success in paternity assignment. Molecular Ecology. 2010;19(7):1512-. doi: 10.1111/j.1365-294X.2010.04544.x 10.1111/j.1365-294X.2007.03089.x 17305863

[51] Rousset F. GENEPOP '007: a complete re-implementation of the GENEPOP software for Windows and Linux. Molecular Ecology Resources. 2008;8(1):103–6. doi: 10.1111/j.1471-8286.2007.01931.x 21585727 10.1111/j.1471-8286.2007.01931.x

[52] Excoffier L, Laval G, Schneider S. Arlequin (version 3.0): an integrated software package for population genetics data analysis. Evolutionary Bioinformatics Online. 2005;1:47. PMC2658868 19325852

[53] Jensen JL, Bohonak AJ, Kelley ST. Isolation by distance, web service. BMC Genetics. 2005;6(1):13. 15760479 10.1186/1471-2156-6-13 PMC1079815

[54] Pritchard JK, Stephens M, Donnelly P. Inference of population structure using multilocus genotype data. Genetics. 2000;155(2):945–59. 10835412 10.1093/genetics/155.2.945 PMC1461096

[55] Jakobsson M, Rosenberg NA. CLUMPP: a cluster matching and permutation program for dealing with label switching and multimodality in analysis of population structure. Bioinformatics. 2007;23(14):1801–6. doi: 10.1093/bioinformatics/btm233 17485429 10.1093/bioinformatics/btm233

[56] R-Core-Team. R: A Language and Environment for Statistical Computing 2012. http://www.R-project.org.

[57] Jombart T. Adegenet: a R package for the multivariate analysis of genetic markers. Bioinformatics. 2008;24(11):1403–5. doi: 10.1093/bioinformatics/btn129 18397895 10.1093/bioinformatics/btn129

[58] Wilson GA, Rannala B. Bayesian inference of recent migration rates using multilocus genotypes. Genetics. 2003;163(3):1177–91. 12663554 10.1093/genetics/163.3.1177 PMC1462502

[59] Antao T, Lopes A, Lopes RJ, Beja-Pereira A, Luikart G. LOSITAN: A workbench to detect molecular adaptation based on a Fst-outlier method. BMC Bioinformatics. 2008;9(1):323 doi: 10.1186/1471-2105-9-323 18662398 10.1186/1471-2105-9-323 PMC2515854

[60] Wondji CS, De Silva WAPP, Hemingway J, Ranson H, Karunaratne SHPP. Characterization of knockdown resistance in DDT- and pyrethroid-resistant *Culex quinquefasciatus* populations from Sri Lanka. Tropical Medicine and International Health. 2008;13(4):548–55. doi: 10.1111/j.1365-3156.2008.02033.x 18312471 10.1111/j.1365-3156.2008.02033.x

[61] Hickner PV, Mori A, Chadee DD, Severson DW. Composite linkage map and enhanced genome map for *Culex pipiens* complex mosquitoes. Journal of Heredity. 2013;104(5):649–55. doi: 10.1093/jhered/est040 23846985 10.1093/jhered/est040 PMC3741631

[62] Platt N, Kwiatkowska RM, Irving H, Diabate A, Dabire R, Wondji CS. Target-site resistance mutations (*Kdr* and *Rdl*), but not metabolic resistance, negatively impact male mating competiveness in the malaria vector *Anopheles gambiae*. Heredity. 2015;115(3):243–52. doi: 10.1038/hdy.2015.33 25899013 10.1038/hdy.2015.33 PMC4519523

[63] Yewhalaw D, Wassie F, Steurbaut W, Spanoghe P, Van Bortel W, Denis L, et al Multiple insecticide resistance: an impediment to insecticide-based malaria vector control program. PLoS One. 2011;6(1). doi: 10.1371/journal.pone.0016066 21264325 10.1371/journal.pone.0016066 PMC3020220

[64] Namountougou M, Simard F, Baldet T, Diabate A, Ouedraogo JB, Martin T, et al Multiple insecticide resistance in *Anopheles gambiae* s.l. populations from Burkina Faso, West Africa. PLoS One. 2012;7(11). doi: 10.1371/journal.pone.0048412 23189131 10.1371/journal.pone.0048412 PMC3506617

[65] Corbel V, N'Guessan R, Brengues C, Chandre F, Djogbenou L, Martin T, et al Multiple insecticide resistance mechanisms in *Anopheles gambiae* and *Culex quinquefasciatus* from Benin, West Africa. Acta Trop. 2007;101(3):207–16. doi: 10.1016/j.actatropica.2007.01.005 17359927 10.1016/j.actatropica.2007.01.005

[66] Pocquet N, Milesi P, Makoundou P, Unal S, Zumbo B, Atyame C, et al Multiple insecticide resistances in the disease vector *Culex p. quinquefasciatus* from Western Indian Ocean. PLoS One. 2013;8(10). doi: 10.1371/journal.pone.0077855 24204997 10.1371/journal.pone.0077855 PMC3804603

[67] Yadouleton A, Badirou K, Agbanrin R, Joest H, Attolou R, Srinivasan R, et al Insecticide resistance status in *Culex quinquefasciatus* in Benin. Parasites & Vectors. 2015;8 doi: 10.1186/s13071-015-0638-3 25582308 10.1186/s13071-015-0638-3 PMC4297371

[68] Nalwanga E, Sempebwa JC. Knowledge and practices of in-home pesticide use: a community survey in Uganda. Journal of Environmental and Public Health. 2011;2011:230894-. doi: 10.1155/2011/230894 . 21776435 10.1155/2011/230894 PMC3136098

[69] Jones CM, Machin C, Mohammed K, Majambere S, Ali AS, Khatib BO, et al Insecticide resistance in *Culex quinquefasciatus* from Zanzibar: implications for vector control programmes. Parasites & Vectors. 2012;5 doi: 10.1186/1756-3305-5-78 22520274 10.1186/1756-3305-5-78 PMC3349604

[70] Kigozi R, Baxi SM, Gasasira A, Sserwanga A, Kakeeto S, Nasr S, et al Indoor residual spraying of insecticide and malaria morbidity in a high transmission intensity area of Uganda. PLoS One. 2012;7(8). doi: 10.1371/journal.pone.0042857 22880123 10.1371/journal.pone.0042857 PMC3412792

[71] Yeka A, Gasasira A, Mpimbaza A, Achan J, Nankabirwa J, Nsobya S, et al Malaria in Uganda: Challenges to control on the long road to elimination I. Epidemiology and current control efforts. Acta Tropica. 2012;121(3):184–95. doi: 10.1016/j.actatropica.2011.03.004 21420377 10.1016/j.actatropica.2011.03.004 PMC3156969

[72] Kudom AA, Mensah BA, Froeschl G, Boakye D, Rinder H. Preliminary assessment of the potential role of urbanization in the distribution of carbamate and organophosphate resistant populations of *Culex* species in Ghana. Parasites & Vectors. 2015;8 doi: 10.1186/s13071-014-0621-4 25566816 10.1186/s13071-014-0621-4 PMC4297417

[73] Kudom AA, Mensah BA, Nunoo J. Assessment of anti mosquito measures in households and resistance status of *Culex* species in urban areas in southern Ghana: Implications for the sustainability of ITN use. Asian Pacific Journal of Tropical Medicine. 2013;6(11):859–64. doi: 10.1016/S1995-7645(13)60153-4 24083580 10.1016/S1995-7645(13)60153-4

[74] Sarkar M, Borkotoki A, Baruah I, Bhattacharyya IK, Srivastava RB. Molecular analysis of knock down resistance (*kdr*) mutation and distribution of *kdr* genotypes in a wild population of *Culex quinquefasciatus* from India. Tropical Medicine & International Health. 2009;14(9):1097–104. doi: 10.1111/j.1365-3156.2009.02323.x 19563477 10.1111/j.1365-3156.2009.02323.x

[75] Ponce G, Rodriguez‐Sanchez IP, Garcia S, Torrado JM, Lozano S, Flores AE. First report of *kdr* mutation (L1014F) in *Culex quinquefasciatus* of México. Insect Science. 2015. 10.1111/1744-7917.12218 25765734

[76] Martinez-Torres D, Chevillon C, Brun-Barale A, Bergé JB, Pasteur N, Pauron D. Voltage-dependent Na+ channels in pyrethroid‐resistant *Culex pipiens* L. mosquitoes. Pesticide Science. 1999;55(10):1012–20.

[77] Sarkar M, Baruah I, Srivastava RB, Borkotoki A, Bhattacharyya IK. High-throughput approach to detection of knockdown resistance (*kdr*) mutation in mosquitoes, *Culex quinquefasciatus*, based on real-time PCR using single-labelled hybridisation probe/melting curve analysis. Pest Management Science. 2011;67(2):156–61. doi: 10.1002/ps.2044 20981727 10.1002/ps.2044

[78] Remnant EJ, Good RT, Schmidt JM, Lumb C, Robin C, Daborn PJ, et al Gene duplication in the major insecticide target site, *Rdl*, in *Drosophila melanogaster*. Proceedings of the National Academy of Sciences of the United States of America. 2013;110(36):14705–10. doi: 10.1073/pnas.1311341110 23959864 10.1073/pnas.1311341110 PMC3767507

[79] Martins WFS, Subramaniam K, Steen K, Mawejje H, Liloglou T, Donnelly MJ, et al Detection and quantitation of copy number variation in the voltage-gated sodium channel gene of the mosquito *Culex quinquefasciatus*. Scientific Reports. 2017;7(1):5821 doi: 10.1038/s41598-017-06080-8 28725028 10.1038/s41598-017-06080-8 PMC5517494

[80] Edi CV, Djogbenou L, Jenkins AM, Regna K, Muskavitch MAT, Poupardin R, et al CYP6 P450 enzymes and *ACE-1* duplication produce extreme and multiple insecticide resistance in the malaria mosquito *Anopheles gambiae*. PLoS Genetics. 2014;10(3). doi: 10.1371/journal.pgen.1004236 24651294 10.1371/journal.pgen.1004236 PMC3961184

[81] Weetman D, Mitchell SN, Wilding CS, Birks DP, Yawson AE, Essandoh J, et al Contemporary evolution of resistance at the major insecticide target site gene *Ace‐1* by mutation and copy number variation in the malaria mosquito *Anopheles gambiae*. Molecular Ecology. 2015;24:2656–72. doi: 10.1111/mec.13197 25865270 10.1111/mec.13197 PMC4447564

[82] Kothera L, Nelms BM, Reisen WK, Savage HM. Population genetic and admixture analyses of *Culex pipiens* complex (Diptera: Culicidae) populations in California, United States. The American Journal of Tropical Medicine and Hygiene. 2013;89(6):1154–67. doi: 10.4269/ajtmh.13-0040 23958909 10.4269/ajtmh.13-0040 PMC3854894

[83] Cartaxo MFS, Ayres CFJ, Weetman D. Loss of genetic diversity in *Culex quinquefasciatus* targeted by a lymphatic filariasis vector control program in Recife, Brazil. Transactions of the Royal Society of Tropical Medicine and Hygiene. 2011;105(9):491–9. doi: 10.1016/j.trstmh.2011.05.004 21737112 10.1016/j.trstmh.2011.05.004

[84] Kawecki TJ, Ebert D. Conceptual issues in local adaptation. Ecology Letters. 2004;7(12):1225–41. doi: 10.1111/j.1461-0248.2004.00684.x

[85] Paris M, Boyer S, Bonin A, Collado A, David J-P, Despres L. Genome scan in the mosquito *Aedes rusticus*: population structure and detection of positive selection after insecticide treatment. Molecular Ecology. 2010;19(2):325–37. doi: 10.1111/j.1365-294X.2009.04437.x 20015143 10.1111/j.1365-294X.2009.04437.x

[86] Kothera L, Godsey MS Jr., Doyle MS, Savage HM. Characterization of *Culex pipiens* complex (Diptera: Culicidae) populations in Colorado, USA using microsatellites. Plos One. 2012;7(10). doi: 10.1371/journal.pone.0047602 23094068 10.1371/journal.pone.0047602 PMC3477124

[87] Mendki MJ, Sharma AK, Veer V, Agrawal OP, Prakash S, Parashar BD. Population genetic structure of *Culex quinquefasciatus* in India by ISSR marker. Asian Pacific Journal of Tropical Medicine. 2011;4(5):357–62. doi: 10.1016/S1995-7645(11)60103-X 21771676 10.1016/S1995-7645(11)60103-X

[88] Wilke ABB, Vidal PO, Suesdek L, Marrelli MT. Population genetics of neotropical *Culex quinquefasciatus* (Diptera: Culicidae). Parasites & Vectors. 2014;7:468-. doi: 10.1186/s13071-014-0468-8 . 25280576 10.1186/s13071-014-0468-8 PMC4190383

[89] Orsini L, Mergeay J, Vanoverbeke J, De Meester L. The role of selection in driving landscape genomic structure of the waterflea *Daphnia magna*. Molecular Ecology. 2013;22(3):583–601. doi: 10.1111/mec.12117 23174029 10.1111/mec.12117

[90] Nabyonga L, Nalwanga S, Buwembo W, Mukasa MK, Kironde F. *Plasmodium falciparum* transmission and insecticide resistance in Iganga, Uganda. Pathogens and Global Health. 2013;107(8):438–9.

