# Supplementary material for: Local selection in the presence of high levels of gene flow: Evidence of heterogeneous insecticide selection pressure across Ugandan Culex quinquefasciatus populations
Source: PLoS Negl Trop Dis. 2017 Oct 3;11(10):e0005917. doi: 10.1371/journal.pntd.0005917 (PMC5640252; doi:10.1371/journal.pntd.0005917)
Supplement: S1 Methods — (PDF) [file pntd.0005917.s001.pdf]

1. Allelic discrimination assays of insecticide target-site mutations in *Culex quinquefasciatus*

1.1 PCR amplification and cloning of genomic region encompassing the *Vgsc*-L1014F and *Ace1*-G119S locus

For the initial design of target-site mutation genotyping assays, partial genomic fragments spanning the location of the *Ace1-G119S* and *Vgsc- L1014F* mutations were amplified from genomic DNA isolated from mosquitoes collected from four different regions (Figure 1). PCR reactions were performed in a final volume of 20 µl including 1µl of genomic DNA, 1X Phusion HF buffer, 200 µM of each dNTP, 0.02 U/µl of Phusion Hot start II DNA polymerase and 0.4 µM of each specific primer for the *Ace-1* and *Vgsc* gene (Table SA). Amplification was performed with cycling conditions of 98 ºC for 30 sec, followed by 30 cycles of 98 ºC for 10 sec, 56 ºC for 15 sec and 72 ºC for 15 sec. with a final extension of 72 ºC for 10 min.

PCR products were purified using the GeneJET PCR purification kit (Thermo Scientific) and then cloned into the pJet 1.2 vector using the CloneJet PCR cloning kit (Thermo Scientific). Clones were screened using PCR and DNA extracted for sequencing from positive colonies using the GeneJET Plasmid Miniprep kit.

After sequencing, sequences were aligned and manually edited in CodonCode Aligner (CodonCode Corporation). Sequence regions conserved across haplotypes were used to design custom primers and TaqMan probes using the Custom TaqMan® Assay Design Tool (Life Technologies, UK) and pyrosequencing assay using the PyroMark assay design software 2.0 (Qiagen).

1.2 *Ace1*-G119S genotyping by Taqman allelic discrimination assay.

Ace1-G119S TaqMan allelic discrimination reactions were carried out in a final volume of 10 µl using 1 µl of genomic DNA, 1X SensiMix II probe (Bioline), 900 mM of

each primer and 200 nM of probes (Table SA). Thermocycling was performed on a Stratagene MX3005P, with cycling parameters of 95 °C for 10 min and 40 cycles of 92 °C for 15 sec and 60 °C for 1 min.

1.3 *Vgsc*-L1014F genotyping using pyrosequencing assay

PCR reactions to amplify a fragment of 105 bp was performed in a total of 25 µl containing 10ng of gDNA, 200 µM of each dNTP, 1X of the 10X PCR buffer, 2.0 mM of $MgCl_{2,}$ 0.6 units of HotStarTaq DNA polymerase (Qiagen) and 0.4 µM of each specific primer (Table SA). After initial denaturation at 95 °C for 15 min, PCR amplification was performed for 40 cycles of 94 °C for 30 sec, 58 °C for 30 sec, and 72 °C for 30 sec, followed by a final extension step at 72 °C for10 min. For genotyping by pyrosequencing, single-stranded PCR products were obtained using the PyroMark Q24 Vacuum Prep Workstation and then used in pyrosequencing reactions performed using the PyroMark Gold Q96 reagent kit (Qiagen). Sequencing primer and dispensation order are described in Table SA.

Table SA: Primers and probes for screening the *Ace1*-G119S and *Vgsc*-L1014F mutations in *Culex quinquefasciatus* mosquitoes

**Locus**	**PCR primers**	**Target-site SNP assay**
		**TaqMan allelic discrimination**
***Ace*-1**	Cx_*Ace*-1-F: 5`- CGACTCGGACCCACTCGT - 3`	Primer F: 5` - TCCAGCGTGGCAGTCC - 3`
	Cx_*Ace*-1-R: 5` - CCTACCTCAGTGCCAGGTTC - 3`	Primer R: 5` -GCCGTCATGCTGTGGATCTT – 3`
		Wild-type/probe: 5` - VIC-AGTAGAAGCCACCCCC - 3`
		SNP/probe: 5` - FAM-AGTAGAAGCTACCCCC - 3`
Kdr		**Pyrosequencing**
	Cx-*Vgsc*-F: 5` - CCTCCCGGACAAGGACCTG - 3`	Primer F: 5` - CTTGGCCACCGTAGTGATAGG - 3`
	Cx-*Vgsc*-R: 5` - GGACGCAATCTGGCTTGTTA -3`	Primer R: 5`Biotin - GCTGTTGGCGATGTTTTGACA - 3`
		Seq. primer: 5` - CCGTAGTGATAGGAAATTT - 3`
		Dispensation: 5`(A/C/T)GTCGTGAGTATTCCAGCGTGAAGTC - 3`
