# Supplementary material for: Local selection in the presence of high levels of gene flow: Evidence of heterogeneous insecticide selection pressure across Ugandan Culex quinquefasciatus populations
Source: PLoS Negl Trop Dis. 2017 Oct 3;11(10):e0005917. doi: 10.1371/journal.pntd.0005917 (PMC5640252; doi:10.1371/journal.pntd.0005917)
Supplement: S2 Methods — (PDF) [file pntd.0005917.s002.pdf]

Development of Microsatellite Multiplex Panels for Population Genetic Analysis in *Culex quinquefasciatus* mosquitoes

1. Materials and Methods

1.1 Microsatellite design

1.1.1 In silico microsatellite isolation and primer design

A total of 180 randomly selected *Culex quinquefasciatus* genome supercontigs were downloaded from VectorBase (www.vectorbase.org) and analyzed using SciRoKo 3.4 (Kofler, Schloetterer, and Lelley 2007) to identify perfect di- or tri- nucleotide motifs with the number of repeats ranging from 5 to 20. For each candidate microsatellite, approximately 200 bp of pre- and post-motif flanking sequence was isolated and target loci with flanking sequences containing additional microsatellites sequences were excluded. To reduce the likelihood of physical linkage, only one microsatellite per supercontig was chosen for primer design using primer 3.0 (Rozen and Skaletsky 2000), targeting primer GC content between 50% and 70% and melting temperature between 60 and 65 °C. All primers were checked for unique binding using BLAST against the *Cx. quinquefasciatus* genome in VectorBase. Only primer pairs from which at least one of the primers was unique in the whole genome were selected for PCR. Furthermore, the primer sequences were BLASTed against the GenBank nucleotide database to ensure that they had not been already described as microsatellite marker previously.

1.1.2 Chromosomal assignment of newly designed microsatellite markers

The current *Cx. quinquefasciatus* genome annotation still lacks chromosome assembly, making accurate prediction of the genomic location of supercontigs problematical. Nevertheless, synteny analysis performed by Arensburger *et al.* (2010) among *Cx. quinquefasciatus, Anopheles gambiae* and *D. melanogaster* chromosomes indicated well correlated chromosome arm architecture among these species as shown in Figure 1A.

Based on the high proportion of *Cx. quinquefasciatus* scaffolds with orthologous syntenic blocks within single *An. gambiae* chromosome arms (Arensburger et al. 2010), the likely chromosomal location of the microsatellite markers designed herein was assigned. The number

of orthologs between *An. gambiae* and the supercontig containing the microsatellite markers was performed using the BioMart tools available at VectorBase (www.vectorbase.org assuming that for each *Cx. quinquefasciatus* supercontig the highest number of counted orthologs within a single *An. gambiae* chromosome indicated genomic synteny between the species. Additional information on chromosome assignment is indicated in Figure 1B and no chromosomal assignments based on synteny disagreed with Fig 1B. To verify the precision of the criteria applied herein to the marker's chromosomal assignment, the same analysis was performed using 11 mapped *Cx. quinquefasciatus* microsatellite described elsewhere (Hickner et al. 2010) .

A)

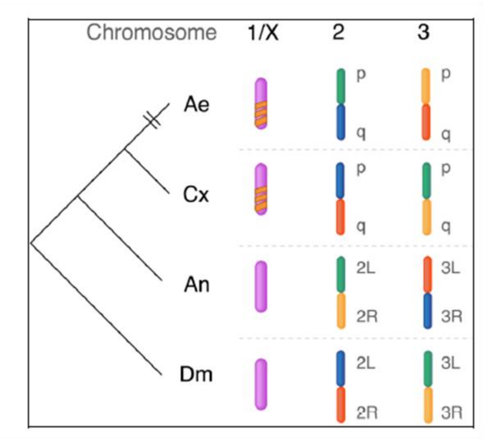

B)

C. quinquefasciatus	*Ae. aegypti*	*An. gambiae*	*D. melanogaster*
Chr.1 p	1	X	X;3R
Chr.1 q	1	2R	3R
Chr.2p	2q	3R	2L
Chr.2q	3q	3L	2R;3L
Chr.3p	2p	2L	3L;2R
Chr.3q	3p	2R	3R (3L)

Reproduced with adaptation from Arensburger et al. (2010)

Figure 1. Chromosomal synteny between *Cx. quinquefasciatus*, *Ae. aegypti*, *An. gambiae* and *D. melanogaster* **A)** Cladogram of *Cx. quinquefasciatus*, *Ae. aegypti*, *An. gambiae* and *D. melanogaster* showing chromosome arm similarities (colours indicate syntenic chromsome arms). The double lines indicate a potential chromosomal arm exchange. **B)** Correlations between *Cx. quinquefasciatus*, *Ae. aegypti*, *An. gambiae* and *D. melanogaster* chromosomes.

1.2 PCR optimization of markers and genetic diversity assessment

1.2.1 Samples

Characterization of all putative microsatellite markers was carried out by genotyping *Cx. quinquefasciatus* samples from two laboratory colonies (CqSF from Recife Brazil (Wilding et al. 2012) and ISOP450 (Hardstone et al. 2007) maintained at the Liverpool School of Tropical Medicine (LSTM) and field mosquitoes from two regions of Uganda (Jinja - N = 35; 00° 25' N, 33°12' E and Kampala – N = 35; 00°20' N, 32°30' E) collected in July 2012. Genomic DNA of individual mosquitoes was isolated using a DNeasy kit (Qiagen). The field samples were confirmed as *Cx. quinquefasciatus* through a diagnostic PCR assay prior to microsatellite genotyping (Smith and Fonseca 2004).

1.2.2 Microsatellite reactions

Initially, thirty-six primer sets were designed and tested individually using eight samples from the two laboratory colonies CqSF (n = 4) and ISOP450 (n = 4). PCR reactions were performed in 25 µl volumes containing 1X buffer, 2mM $MgCl_2$, 0.2mM of each dNTP, 1.25 units of *Taq* polymerase (Fermentas), 0.5 µM of each primer and 2 µl of genomic DNA. The PCR conditions consisted of denaturation for 3 min at 95°C, followed by 30 cycles of 30 s at 95 °C, 30 s at 55 °C, and 30 s at 72 °C, and a final extension at 72 °C for 10 min. PCR products were separated on 2% agarose gels in 1X TAE buffer, stained with ethidium bromide and visualized using UV light. PCR reactions that showed non-specific fragments at the initial conditions were optimized by iteratively increasing the annealing temperature in steps of 2 °C up to a maximum of 65 °C.

1.2.3 Mutiplex development and amplification

Following initial PCR optimization, 30 microsatellite primer pairs that consistently amplified single fragments of the expected fragment size (within the resolution of an agarose gel) in all samples of both laboratory colonies were tested for compatibility in PCR multiplex reactions using Multiplex Manager 1.0 (Holleley and Geerts 2009). Multiplex panels were selected to have 6 loci per reaction, three fluorescent dyes, fragment size differences of at least 30 bp between loci labeled with the same dye and a complementary threshold of 15 (maximum number of AT or CG matches between two primers) to prevent potential hairpins, or dimers between any primers within each reaction. Forward primers of each set were labeled with Beckman-Coulter D2, D3 or D4 fluorescent dyes; however, D3 and D4 were preferentially selected due to their higher signal intensity.

Multiplexes were amplified in 25 µl reaction volumes that included 1X Type-it multiplex PCR Master Mix (Qiagen), 0.2 µM of each primer and 2 µl of genomic DNA. Amplifications were carried out under the following conditions: initial heat activation for 5 min at 95 °C followed by 24 cycles of 30 s at 95°C, 90 s at 60°C, 30 s at 72°C and a final extension step of 30 min at 60°C. For fragment analysis, 2 µl of PCR multiplex reaction were added to a mix of 37.5 µl of Sample Loading Solution and 0.5 µl of 400-bp size Standard (Beckman-Coulter) followed by a denaturation step of 5 min 95°C before fragment analysis on Beckman-Coulter CEQ8000. Genotypes were scored by allelic size using Beckman-Coulter CEQ 2000 DNA analysis system software and manually verified.

1.2.4 Microsatellite markers genetic profiling

Observed and expected heterozygosities were calculated using *GenAlEx* (Peakall and Smouse 2012). Polymorphism information content (PIC) was calculated using CERVUS v3.0.3 (Kalinowski, Taper, and Marshall 2007). Linkage disequilibrium among marker pairs and deviation from Hardy-Weinberg equilibrium for each locus were assessed using the exact tests in GENEPOP (Rousset 2008). The influence of stuttering and large allele dropout on genotypes was analyzed using Micro-Checker (Van Oosterhout et al. 2004).

2. Results

2.1 Chromosomal assignment

Using 11 previously mapped microsatellites to validate the criteria applied here for assignment of markers to chromosomes, our analysis predicted the same chromosome location compared to the published data in 81.81% of the markers tested (Table 1). Only for marker C65AC1 did the map location not correspond to that expected (chromosome 2), with our analysis predicting the likely location to be chromosome 1 or 3. For the marker C44CAC2 mapped at chromosome 3, our analysis could not precisely differentiate the location between chromosome 1 and 3 due to chromosome arm exchange of *Anopheles* chromosome 2R (see Figure 1B).

From the 30 newly designed markers the majority (36.66%) was mapped to chromosome 2, while 6.66 and 16.66% were assigned to chromosome 3 and 1, respectively (Table 2). For 20%

of the markers the likely chromosomal location was either chromosome 1 or 3, but it was not possible to pinpoint these to a specific chromosome. For the other 20% of markers it was not possible to identify the likely chromosomal location due to the absence of orthologous gene(s) in *An. gambiae* (Table 2).

Table 1. Comparison between chromosomal assignment of published microsatellite loci based on published data and on our *in silico* prediction (see text for details)
[a] sourced from Arensburger et al. (2010)

**Published markers with assigned chromosome** [a]				***In silico* chromosome assignment**			
**Marker** [b]	**Genetic locus**	**GenBank accession**	**Map location**	**Number of *Cx. quinquefasciatus* genes nearby the microsatellite marker**	**Number of *An. gambiae* orthologous gene(s)**	**number of orthologous genes between *Cx. quinquefasciatus* and *An. gambiae* / (chromosome assignment)**	***Cx. quinquefasciatus* chromosomal assignment**
C177CA1	CX114	FD664728	2-76.4	17	20	15(3L)	2
C68GA1	LF335	BM005505	2-9.6	25	2	2(3R)	2
C127TC1	CX60	FD664718	1-0.0	31	20	13(x)	1
C99TGT1	CX112	FD664727	3-17.9	18	18	15(2L)	3
C65AC1	CX90	FD664719	2-15.9	30	31	24(2R)	1 or 3
C205TG1	CX17	FD664699	3-18.5	7	5	5(2L)	3
C134AC1	CX61	FD664712	2-42.3	7	1	1(3R)	2
C48GTT1	CX40	FD664709	2-29.2	51	50	45(3R)	2
C32AC1	LF334	BM005506	2-00.0	22	4	4(3R)	2
C139TG1	CX53	FD664714	3-26.0	24	4	4(2L)	3
C446AC2	CX11	FD664697	3-65.8	19	18	18(2R)	1 or 3

[b] microsatellite locus isolated by Hickner et al. (2010)

Table 2. Chromosomal assignment of 30 newly designed *Cx. quinquefasciatus* microsatellite markers

Cx. quinquefasciatus marker name	GenBank accession(s)	*Cx. quinquefasciatus* supercontig	number of orthologous genes between *Cx. quinquefasciatus* and *An. gambiae* / (chromosome assignment)	*An. gambiae* orthologous chromosome	*Cx. quinquefasciatus* chromosomal assignment
MCQ1	GF112161	supercont3.181	13(2R), 3(3R), 2(2L)	2R	1q or 3q
MCQ2	GF112142	supercont3.5	7(3R), 1(3L)	3R	2p
MCQ3	GF112143	supercont3.573	0	-	-
MCQ4	GF112162	supercont3.1082	1(3L)	3L	2q
MCQ5	GF112144	supercont3.1163	1 (3L)	3L	2q
MCQ8	GF112163	supercont3.487	6 (2R)	2R	1q or 3q
MCQ9	GF112164	supercont3.918	0	-	-
MCQ10	GF112145	supercont3.741	2 (3R)	3R	2p
MCQ11	GF112146	supercont3.526	20 (2R)	2R	1q or 3q
MCQ13	GF112165	supercont3.577	1 (2R)	2R	1q or 3q
MCQ16	GF112169	supercont3.627	0	-	-
MCQ19	GF112166	supercont3.78	7 (3R)	3R	2p
MCQ20	GF112147	supercont3.1407	0	-	-
MCQ21	GF112167	supercont3.186	32 (3L)	3L	2q
MCQ22	GF112148	supercont3.792	8(3L), 1(2L)	3L	2q
MCQ23	GF112149	supercont3.91	11(X), 2(3L), 1(2L), 1(2R)	X	1p
MCQ24	GF112168	supercont3.264	13(x), 1(3L), 1(2L)	X	1p
MCQ25	GF112150	supercont3.98	30(3L), 1(2L)	3L	2q
MCQ26	GF112151	supercont3.733	2 (3L)	3L	2q
MCQ28	GF112170	supercont3.138	3(3L), 1(2R)	3L	2q
MCQ29	GF112171	supercont3.1003	2(2L)	2L	3p
MCQ31	GF112152	supercont3.1208	0	-	-
MCQ33	GF112153	supercont3.132	7(x)	X	1p
MCQ34	GF112154	supercont3.147	17(2R), 1(x)	2R	1q or 3q
MCQ36	GF112155	supercont3.337	20 (3R)	3R	2p
MCQ37	GF112156	supercont3.1015	4 (X)	X	1p
MCQ39	GF112157	supercont3.234	2(2R), 15(X), 2(3R), 2(3L)	X	1p

MCQ41	GF112158	supercont3.701	7 (2L)	2L	3p
MCQ42	GF112159	supercont3.1035	0	-	-
MCQ45	GF112160	supercont3.19	24(2R), 1(3R), 1(3L)	2R	1q or 3p

2.2 Microsatellite genetic pattern

Scanning of 180 *Cx. quinquefasciatus* supercontigs identified 214 putative microsatellite sequences with di- and tri- nucleotides repeats. Thirty six perfect di- or tri-nucleotide, single-locus repeats were selected for primer design and characterization. Initial screening of the designed primers through agarose gel electrophoresis showed 30 of the primer pairs with consistent amplification, while the remaining six primer sets were discarded due to either no amplification (MCQ 30, MCQ 40 and MCQ 43) or amplification of non-target regions (MCQ 7, MCQ 17 and MCQ 44) under different reaction and amplification conditions. BLAST analysis against the GenBank nucleotide database of all markers revealed that one locus (MCQ 16), despite differing in primer sequences, matched a microsatellite region previously described (GenBank Accession number DQ388495.1).

Thirty loci were selected for multiplexing in five six-plex reactions (Table 3), which all amplified under the same PCR conditions. All multiplex panels were free from spectral overlap, allele overlap, and excess peaks (Figure 1). Polymorphism and reproducibility of the microsatellite markers was surveyed in 70 *Cx. quinquefasciatus* individuals collected in Uganda. Amplification success was 100% for 21 of the 30 markers, while for the remaining nine loci amplification was between 77.14% and 98.6%. The lowest success rate (77.14%) was observed in multiplex 5 primarily as a result of amplification failure of loci MCQ33 and MCQ34 (Figure 2).

All 30 loci were polymorphic (0.95 criterion), with 2-12 alleles observed per locus (average 5.367 SD 0.388), while the observed and expected mean heterozygosities were 0.484 (SD 0.029) and 0.585 (SD 0.022) respectively (Table 3). Four out of 5 panels exhibited the same proportion of alleles (mean = 5.74), while the mean expected heterozygosity in reaction 2, reaction 4 and reaction 5 was higher than 0.6 (Figure 2). Furthermore, polymorphism information content (PIC) also showed a high level of genetic variability ranging from 0.337 (MCQ 33) to 0.826 (MCQ 26) (Table 1).

Samples from Jinja and Kampala exhibited seven and five significant deviations from Hardy-Weinberg equilibrium (HWE) respectively after Bonferroni correction (Table 4), with four markers (MCQ 28, MCQ 23, MCQ 33 and MCQ 34) showing significant HWE deviation in both populations. For these loci the HWE deviation was associated with positive F_{IS} values (between 0.271 and 0.795) reflecting heterozygote deficiency, probably due to the presence of null alleles. Results from the Fisher's exact test for linkage disequilibrium (Table 5) indicated significant linkage disequilibrium between only loci MCQ 26 and MCQ 31

(Jinja) and MCQ 26 and MCQ 41 (Kampala), suggesting broad-scale independence of markers.

Table 3. Multiplex panels and characteristics of polymorphic microsatellites in Ugandan field populations of *Culex quinquefasciatus*

Multiplex	Locus	Primer sequences (5`-3`)	Dye	Repeat motif	size range (bp)	No of alleles	PIC	*R*s	H_E	GenBank accession no.
Reaction 1	MCQ24	F: CTTCTAGGGTTGGCGTTGAG<br>R: TCGTGATCTGCATCGGTAAT	[D4]	$(AC)_8$	127-139	6	0.653	5.983	0.697	GF112168
	MCQ16	F: ACGCCGTCAGAAACACATTA<br>R: GAGCTGTGTGACAAGCATGG	[D4]	$(AC)_{13}$	197-209	7	0.696	7.000	0.725	GF112169
	MCQ19	F: GTCCCAGGATCGTAGCTCAG<br>R: CGTTCCCCTAGAGTTGGTTG	[D4]	$(GT)_8$	250-261	9	0.563	8.596	0.595	GF112166
	MCQ45	F: GCGCTAGGGACCATACACAA<br>R: TGGTTTCTAACTGGGGCATC	[D3]	$(AG)_{14}$	100-115	8	0.589	7.700	0.633	GF112160
	MCQ3	F: GGGCCAGAGGAAAGTGAGAT<br>R: CCCGAAACCTTAAGCACATC	[D3]	$(GT)_8$	206-214	7	0.454	6.910	0.479	GF112143
	MCQ25	F: CTAGAGGAAAAGCGGTGCAT<br>R: CTGGCTGTCCACACATCAAT	[D3]	$(GT)_{10}$	254-266	5	0.463	4.985	0.545	GF112150
Reaction 2										
	MCQ28	F: TTTGGCAAATAGCCTTCTGG<br>R: GGTGTCGATGTGAGGGGTTA	[D4]	$(TG)_8$	116-126	4	0.553	4.000	0.611	GF112170
	MCQ39	F: TCTTTAGCAGCGCCTGTATG<br>R: ACAACACGTAACCTCGCTGA	[D4]	$(GT)_9$	186-200	7	0.527	6.634	0.566	GF112157
	MCQ10	F: AGCGAGTGCGACGAATAAAG<br>R: CAGCGTGGAAGACAAGTTCA	[D4]	$(CGA)_8$	306-320	8	0.652	7.642	0.693	GF112145
	MCQ2	F: TGATTGAGTCATGGTGCAGAG<br>R: ACCCTTAAGCCTTCCGTGTA	[D3]	$(AG)_{20}$	135-155	3	0.332	2.884	0.412	GF112142
	MCQ4	F: TTCATCTCTATCCGGTTGTGG<br>R: CTAGCCTGGCAAGAACCAAC	[D3]	$(TG)_8$	258-262	4	0.544	3.642	0.621	GF112162
	MCQ29	F: CCCTTGTGGGAAGTAGTTGG<br>R:TGACACTTCCTCGAGACACG	[D2]	$(AC)_8$	119-127	5	0.610	4.909	0.670	GF112171
Reaction 3										

	MCQ1	F: TACCGAGAGGTTTGCAGGAC<br>R: GCGTACCAGGATCGTCTCAT	[D4]	$(AG)_{12}$	133-145	4	0.418	4.000	0.492	GF112161
	MCQ22	F: TCAAATCTGGGTCACAATGC<br>R: TGACAACTTCCCCAGAGAGG	[D4]	$(GT)_{17}$	179-215	9	0.688	8.873	0.731	GF112148
	MCQ37	F: AATCCAAACGACGCAAGAAC<br>R: GGCGCTAGAAGTAGCCTTCA	[D4]	(AC)8	245-251	4	0.361	4.000	0.391	GF112156
	MCQ36	F: GGATGGACTCCACGAAATGT<br>R: GGGTTTCATGGTGTCTACGG	[D3]	$(CA)_{8}$	148-152	4	0.355	3.642	0.425	GF112155
	MCQ23	F: TCTTTAGCTTGCAGGGCCTA<br>R: TTCTGACAGCTGCACTCACC	[D3]	$(CT)_{8}$	244-251	5	0.436	4.600	0.510	GF112149
	MCQ11	F: CATGAGCACGTGTCTTCTCC<br>R: CATCATAATCGGCGCCTAAC	[D2]	$(TG)_{8}$	251-265	7	0.493	6.581	0.566	GF112146
Reaction 4										
	MCQ8	F: CCCCAACATTAAACCCTCTTT<br>R: TTTCTGTACACCTCGCACCA	[D4]	$(CA)_{13}$	213-221	5	0.604	4.642	0.667	GF112163
	MCQ9	F: TTAGCGGAGCAGCTGTGTAG<br>R: GTGCCTCAAGAGTCCATCGT	[D4]	$(GA)_{10}$	271-281	6	0.665	5.650	0.720	GF112164
	MCQ31	F: AATGAAGGAACCTCGCGTAA<br>R: ATTTAGATGCGACCGCAGAA	[D4]	$(TG)_{8}$	158-170	6	0.717	5.642	0.756	GF112152
	MCQ42	F: AAGGGTCACAACCCACTTCA<br>R: TGGTGGGGACACATGTTAAA	[D3]	$(CT)_{13}$	122-136	8	0.548	7.636	0.582	GF112159
	MCQ13	F: ACAGAGCTGCCTTTTGCAGT<br>R: CCAGCTGCCAATTTCATTCT	[D3]	$(AC)_{9}$	248-256	6	0.602	5.91	0.664	GF112165
	MCQ41	F: GAAGGCACTTCCTGTCTCGT<br>R:TGGTCGTAACAATCCCCTT	[D3]	$(AG)_{8}$	194-202	6	0.651	5.989	0.709	GF112158
Reaction 5										
	MCQ33	F: ATCCGCTCGACAAATAATGG<br>R: ACATGGAACGACGTCGAAAT	[D4]	$(CA)_{9}$	110-114	4	0.368	4.000	0.410	GF112153
	MCQ20	F: TGTATGATGCTGTGCGATGA<br>R: CAAACCTTGCCAAAGAGTGC	[D4]	$(AG)_{9}$	198-202	3	0.554	3.000	0.626	GF112147
	MCQ21	F: CAGCTCGGCAATAGAAAACC<br>R: TCTGTCTCTGTCTGCCTTGC	[D4]	$(CT)_{10}$	241-271	12	0.617	11.14	0.652	GF112167
	MCQ26	F: ACCTGTCACTCGAGCCATTC<br>R: GTGCGACATCCGATACTGAA	[D4]	$(CT)_{9}$	145-167	10	0.825	9.873	0.844	GF112151

MCQ34	F: CAGTGGGGAAAGAAACCAGA<br>R: TTAAGCCAATCTGCGTTGTG	[D3]	$(CA)_9$	172-190	8	0.625	7.541	0.674	GF112154
MCQ5	F: GGAAATCCATTGGACAGGAA<br>R: ACATCGTCGGAGGAACAGAG	[D2]	$(AC)_9$	198-206	6	0.641	5.640	0.694	GF112144

Polymorphism Information content (PIC), Allelic richness (*R*s) and expected heterozygosity (*H*E).

Table 4. Test for departure from Hardy-Weinberg (Fisher's exact test) and inbreeding coefficient (F_{IS}).

		Population			
		Jinja		Kampala	
**Multiplex**	**Locus**	P [a]	F_{IS} [b]	P [a]	F_{IS} [b]
Reaction 1	MCQ24	0.004	0.108	0.445	-0.069
	MCQ16	0.457	0.094	0.138	0.127
	MCQ19	0.890	-0.052	0.003	0.119
	MCQ45	0.003	-0.062	0.181	0.056
	MCQ3	0.015	0.240	0.002	0.334
	MCQ25	0.001 *	0.386	0.055	0.272
Reaction 2					
	MCQ28	0.000*	0.658	0.000*	0.488
	MCQ39	0.245	0.090	1.000	-0.067
	MCQ10	0.000*	0.398	0.134	0.221
	MCQ2	0.717	0.060	0.002	0.328
	MCQ4	0.647	0.083	0.677	0.020
	MCQ29	0.645	-0.127	0.090	0.158
Reaction 3					
	MCQ1	0.000*	0.504	0.002	0.415
	MCQ22	0.013	0.359	0.424	0.060
	MCQ37	0.694	0.102	0.249	-0.097
	MCQ36	0.860	-0.087	1.000	-0.019
	MCQ23	0.000*	0.598	0.000*	0.367
	MCQ11	0.614	-0.022	0.399	-0.017
Reaction 4					

	MCQ8	0.218	0.221	0.132	0.066
	MCQ9	0.759	0.058	0.046	0.190
	MCQ31	0.972	-0.107	0.132	-0.015
	MCQ42	0.869	-0.036	0.046	-0.053
	MCQ13	0.398	0.175	0.523	0.123
	MCQ41	0.707	-0.068	0.288	0.024
Reaction 5					
	MCQ33	0.000*	0.795	0.001*	0.465
	MCQ20	0.513	0.129	0.006	0.355
	MCQ21	0.000*	0.414	0.079	0.208
	MCQ26	0.465	-0.033	0.236	0.103
	MCQ34	0.001*	0.409	0.000*	0.271
	MCQ5	0.043	0.230	0.001*	0.370

Departure from Hardy-Weinberg after correction for multiple test (adjusted significance [5%] threshold = 0.001666); *Significant.

[a] Probability values using Fisher`s method.

[b] Weir and Cockerham's (1984) inbreeding coefficient.

Table 5. Linkage disequilibrium test in samples collected in Jinja (below diagonal) and Kampala (above diagonal)

	MCQ24	MCQ16	MCQ19	MCQ 45	MCQ 3	MCQ 25	MCQ 28	MCQ 39	MCQ 10	MCQ 2	MCQ 4	MCQ 29	MCQ 1	MCQ 22	MCQ 37	MCQ 36	MCQ 23	MCQ 11	MCQ 8	MCQ 9	MCQ 31	MCQ42	MCQ 13	MCQ41	MCQ 33	MCQ 20	MCQ21	MCQ26	MCQ34	MCQ5
MCQ24	*																													
MCQ16		*																												
MCQ19			*																											
MCQ45				*																										
MCQ3					*																									
MCQ25						*																								
MCQ28							*																							
MCQ39								*																						
MCQ10									*																					
MCQ2										*																				
MCQ4											*																			
MCQ29												*																		
MCQ1													*																	
MCQ22														*																
MCQ37															*															
MCQ36																*														
MCQ23																	*													
MCQ11																		*												
MCQ8																			*											
MCQ9																				*										
MCQ31																					*									
MCQ42																						*								
MCQ13																							*							
MCQ41																								*				§		
MCQ33																									*					
MCQ20																										*				
MCQ21																											*			
MCQ26																					§							*		
MCQ34																													*	
MCQ5																														*

Linkage disequilibrium after correction for multiple test (adjusted significance [5%] threshold = 0.001666), §; significant.

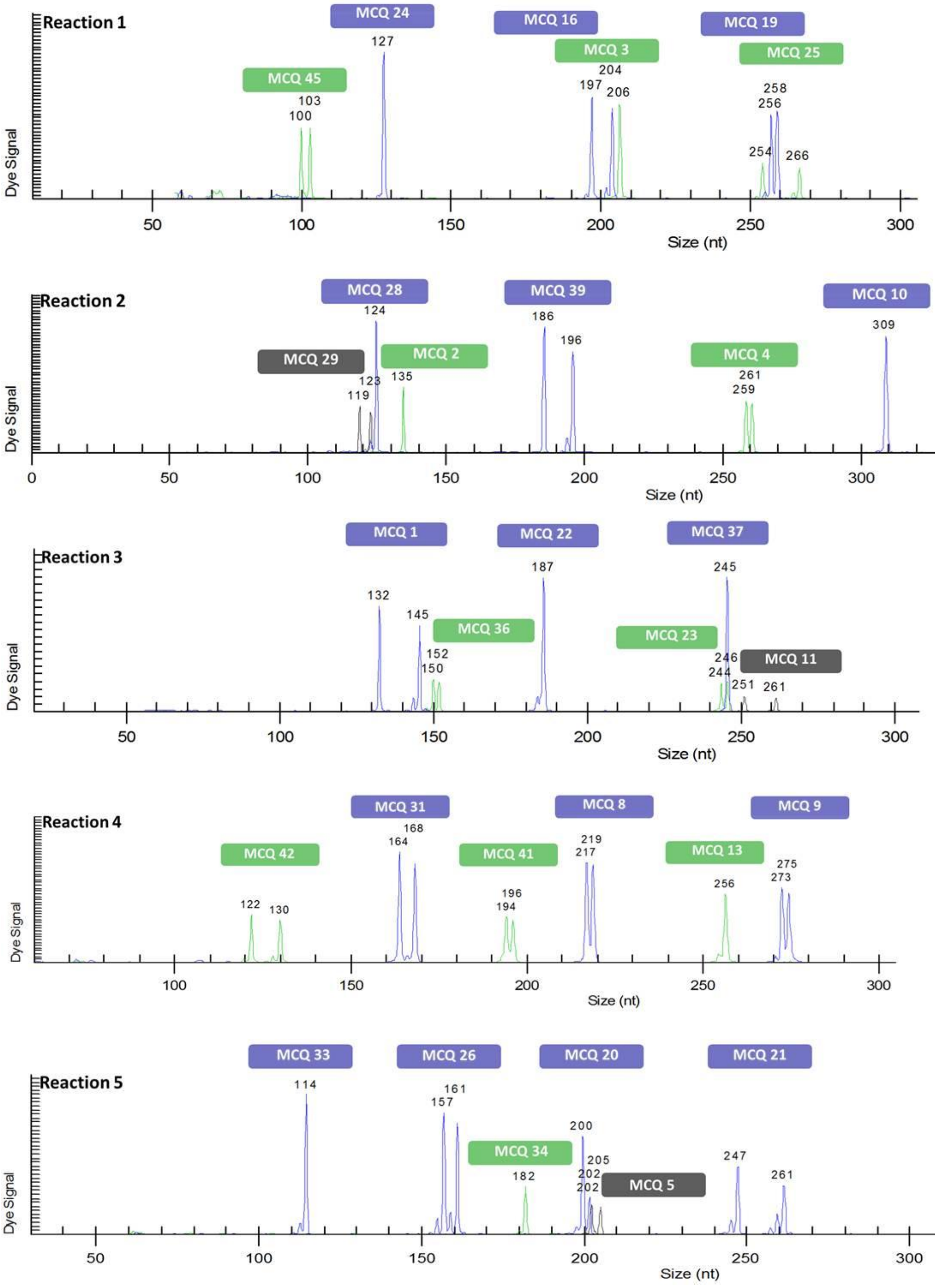

Figure 1. Electropherograms of the five multiplex panels depicting representative amplification profiles and loci distribution. Horizontal axis shows the size in base pairs (bp). Numbers above the peaks are the allele size (bp). Black, green and blue labels correspond to fluorescent signal of the dyes D2, D3 and D4 respectively.

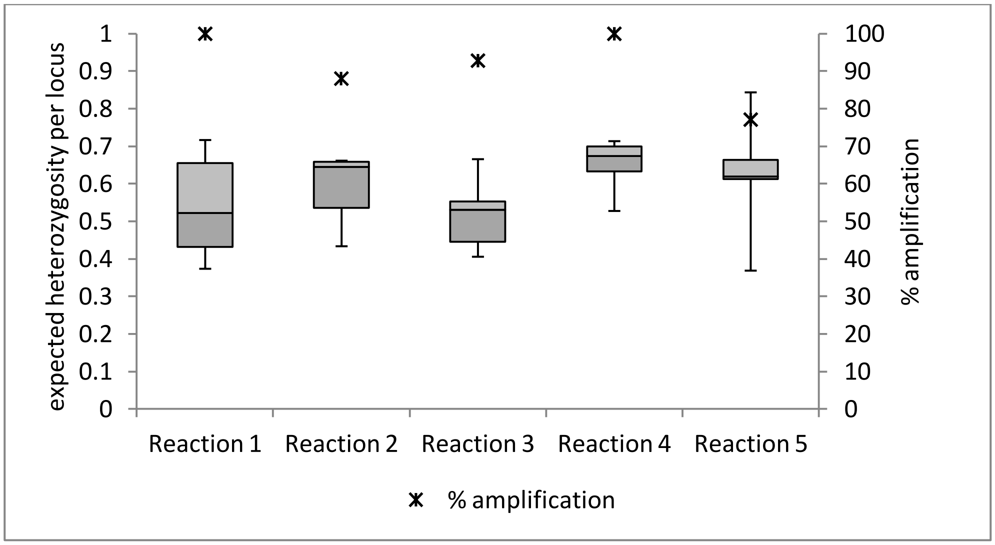

Figure 2. Proportion of amplification and expected heterozygosity in each six-plex panel. % amplification: proportion of samples per reaction that amplified for all loci. Box plot includes expected heterozygosity from the 25th to the 75th percentile; the horizontal line within the box represents the median value. Lines outside the box represent the lowest and highest value.

3. References

Arensburger, Peter, Karine Megy, Robert M. Waterhouse, Jenica Abrudan, Paolo Amedeo, Beatriz Antelo, Lyric Bartholomay, Shelby Bidwell, Elisabet Caler, Francisco Camara, Corey L. Campbell, Kathryn S. Campbell, Claudio Casola, Marta T. Castro, Ishwar Chandramouliswaran, Sinead B. Chapman, Scott Christley, Javier Costas, Eric Eisenstadt, Cedric Feschotte, Claire Fraser-Liggett, Roderic Guigo, Brian Haas, Martin Hammond, Bill S. Hansson, Janet Hemingway, Sharon R. Hill, Clint Howarth, Rickard Ignell, Ryan C. Kennedy, Chinnappa D. Kodira, Neil F. Lobo, Chunhong Mao, George Mayhew, Kristin Michel, Akio Mori, Nannan Liu, Horacio Naveira, Vishvanath Nene, Nam Nguyen, Matthew D. Pearson, Ellen J. Pritham, Daniela Puiu, Yumin Qi, Hilary Ranson, Jose M. C. Ribeiro, Hugh M. Roberston, David W. Severson, Martin Shumway, Mario Stanke, Robert L. Strausberg, Cheng Sun, Granger Sutton, Zhijian Tu, Jose Manuel C. Tubio, Maria F. Unger, Dana L. Vanlandingham, Albert J. Vilella, Owen White, Jared R. White, Charles S. Wondji, Jennifer Wortman, Evgeny M. Zdobnov, Bruce Birren, Bruce M. Christensen, Frank H. Collins, Anthony Cornel, George Dimopoulos, Linda I. Hannick, Stephen Higgs, Gregory C. Lanzaro, Daniel Lawson, Norman H. Lee, Marc A. T. Muskavitch, Alexander S. Raikhel, and Peter W. Atkinson. 2010. Sequencing of Culex quinquefasciatus Establishes a Platform for Mosquito Comparative Genomics. *Science* 330 (6000):86-88.

Hardstone, Melissa C., Cheryl Leichter, Laura C. Harrington, Shinji Kasai, Takashi Tomita, and Jeffrev G. Scott. 2007. Cytochrome P450 monooxygenase-mediated permethrin resistance confers limited and larval specific cross-resistance in the southern house mosquito, Culex pipiens quinquefasciatus. *Pesticide Biochemistry and Physiology* 89 (3):175-184.

Hickner, Paul V., Becky deBruyn, Diane D. Lovin, Akio Mori, Susanta K. Behura, Robert Pinger, and David W. Severson. 2010. Genome-Based Microsatellite Development in the Culex pipiens Complex and Comparative Microsatellite Frequency with Aedes aegypti and Anopheles gambiae. *Plos One* 5 (9).

Holleley, Clare E. and Paul G. Geerts. 2009. Multiplex Manager 1.0: a cross-platform computer program that plans and optimizes multiplex PCR. *Biotechniques* 46 (7):511-+.

Kalinowski, Steven T., Mark L. Taper, and Tristan C. Marshall. 2007. Revising how the computer program CERVUS accommodates genotyping error increases success in paternity assignment. *Molecular Ecology* 16 (5):1099-1106.

Kofler, Robert, Christian Schloetterer, and Tamas Lelley. 2007. SciRoKo: a new tool for whole genome microsatellite search and investigation. *Bioinformatics* 23 (13):1683-1685.

Peakall, Rod and Peter E. Smouse. 2012. GenAlEx 6.5: genetic analysis in Excel. Population genetic software for teaching and research-an update. *Bioinformatics* 28 (19):2537-2539.

Rousset, Francois. 2008. GENEPOP ' 007: a complete re-implementation of the GENEPOP software for Windows and Linux. *Molecular Ecology Resources* 8 (1):103-106.

Rozen, S. and H. Skaletsky. 2000. Primer3 on the WWW for general users and for biologist programmers. *Methods in molecular biology (Clifton, N.J.)* 132:365-86.

Smith, J. L. and D. M. Fonseca. 2004. Rapid assays for identification of members of the Culex (Culex) pipiens complex, their hybrids, and other sibling species (Diptera: Culicidae). *The American journal of tropical medicine and hygiene* 70 (4):339-345.

Van Oosterhout, C., W. F. Hutchinson, D. P. M. Wills, and P. Shipley. 2004. MICRO-CHECKER: software for identifying and correcting genotyping errors in microsatellite data. *Molecular Ecology Notes* 4 (3):535-538.

Weir, B. S. and C. C. Cockerham. 1984. ESTIMATING F-STATISTICS FOR THE ANALYSIS OF POPULATION-STRUCTURE. *Evolution* 38 (6):1358-1370.

Wilding, Craig S., Ian Smith, Amy Lynd, Alexander Egyir Yawson, David Weetman, Mark J. I. Paine, and Martin J. Donnelly. 2012. A cis-regulatory sequence driving metabolic insecticide resistance in mosquitoes: Functional characterisation and signatures of selection. *Insect Biochemistry and Molecular Biology* 42 (9):699-707.
