# Supplementary material for: Local selection in the presence of high levels of gene flow: Evidence of heterogeneous insecticide selection pressure across Ugandan Culex quinquefasciatus populations
Source: PLoS Negl Trop Dis. 2017 Oct 3;11(10):e0005917. doi: 10.1371/journal.pntd.0005917 (PMC5640252; doi:10.1371/journal.pntd.0005917)
Supplement: S1 Fig — (PDF) [file pntd.0005917.s007.pdf]

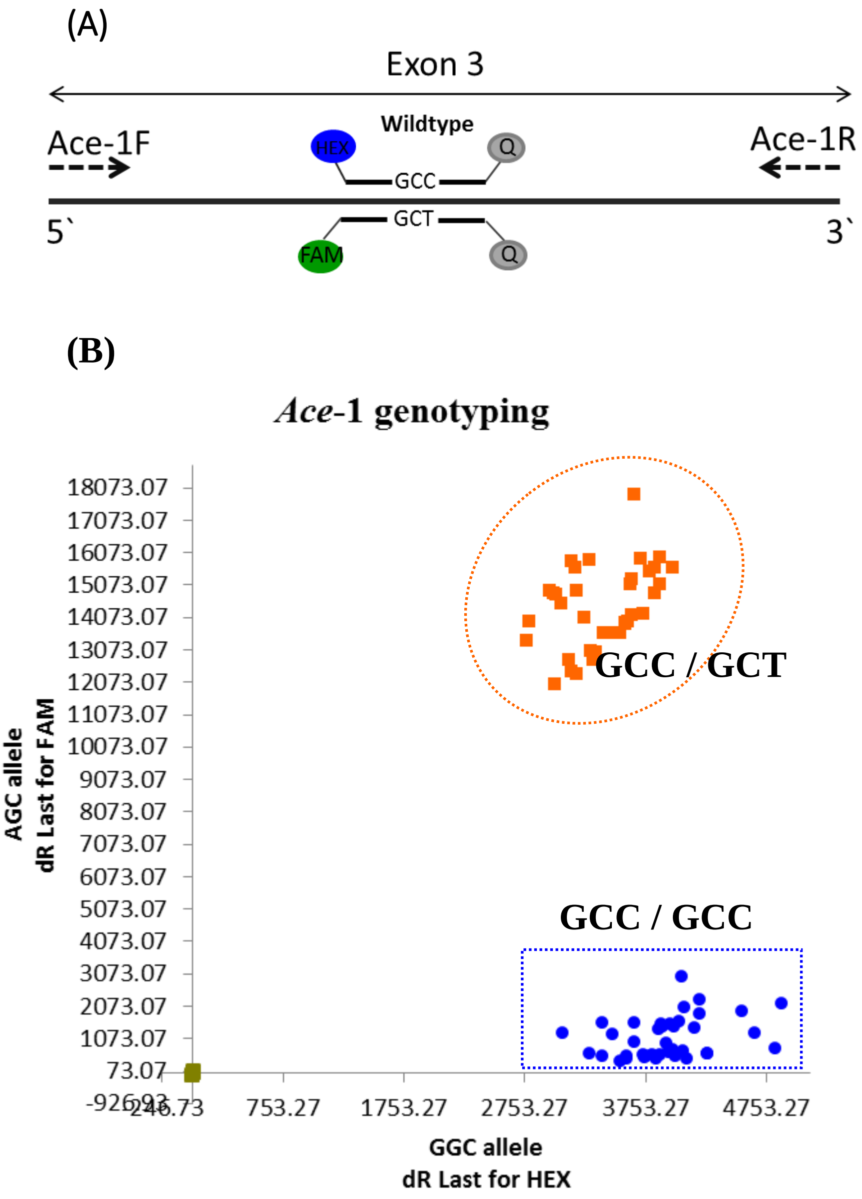

Figure S1. *Ace*-1 target-site mutation (G119S) genotyping in *Cx. quinquefasciatus*. A) Scheme of the TaqMan probes for 119S allelic discrimination. Susceptible allele-specific probe labelled with HEX and resistant allele-specific probe labelled with 6-FAM. B) Allelic discrimination for the 119 codon position. X-axis designates the susceptible allele and the y-axis designates the resistant allele. Blue dots indicate individuals homozygous for the wild-type and orange squares indicates individuals heterozygous for the mutation.
