# Supplementary material for: Local selection in the presence of high levels of gene flow: Evidence of heterogeneous insecticide selection pressure across Ugandan Culex quinquefasciatus populations
Source: PLoS Negl Trop Dis. 2017 Oct 3;11(10):e0005917. doi: 10.1371/journal.pntd.0005917 (PMC5640252; doi:10.1371/journal.pntd.0005917)
Supplement: S2 Fig — (PDF) [file pntd.0005917.s008.pdf]

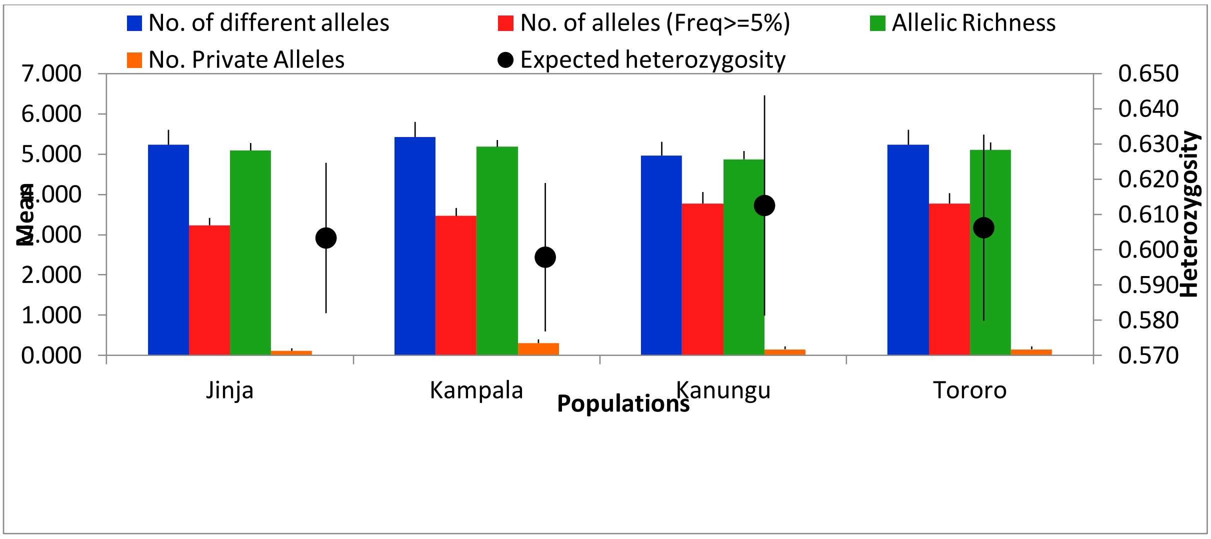


**Figure S2.** Genetic diversity estimates across Ugandan *Cx. quinquefasciatus* populations based on 26 microsatellite markers. Allelic richness was calculated based on sample size of N=33. No=Number of different alleles, No (Freq >=5%)=Number of different alleles with a frequency >=5%, No. Private alleles = Number of alleles unique to a single population.
