# Supplementary material for: Local selection in the presence of high levels of gene flow: Evidence of heterogeneous insecticide selection pressure across Ugandan Culex quinquefasciatus populations
Source: PLoS Negl Trop Dis. 2017 Oct 3;11(10):e0005917. doi: 10.1371/journal.pntd.0005917 (PMC5640252; doi:10.1371/journal.pntd.0005917)
Supplement: S3 Fig — (PDF) [file pntd.0005917.s009.pdf]

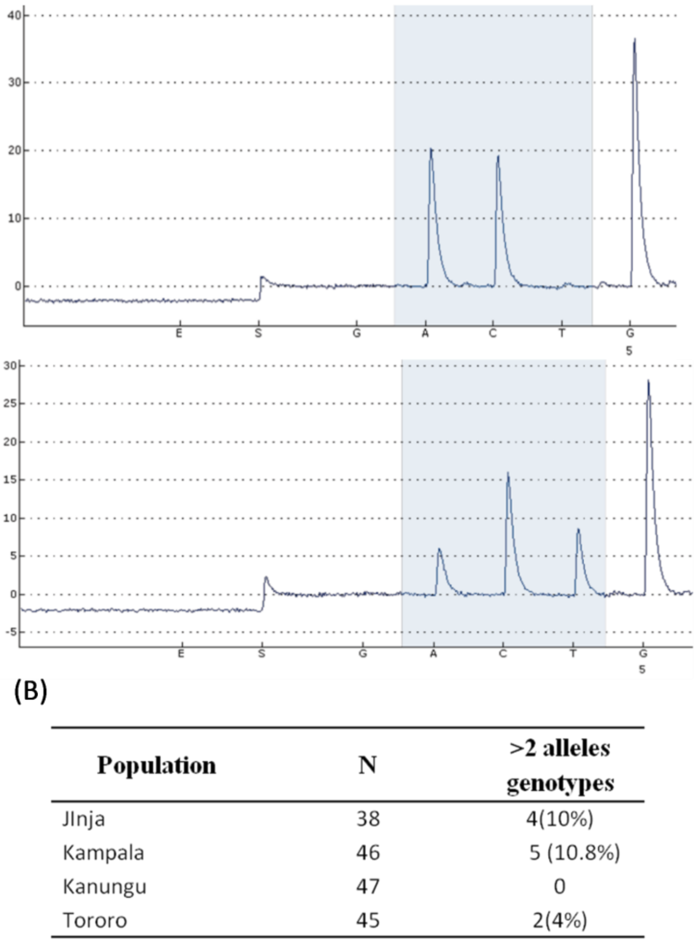


(B)

| Population | N | >2 alleles genotypes |
|---|---|---|
| JInja | 38 | 4(10%) |
| Kampala | 46 | 5 (10.8%) |
| Kanungu | 47 | 0 |
| Tororo | 45 | 2(4%) |

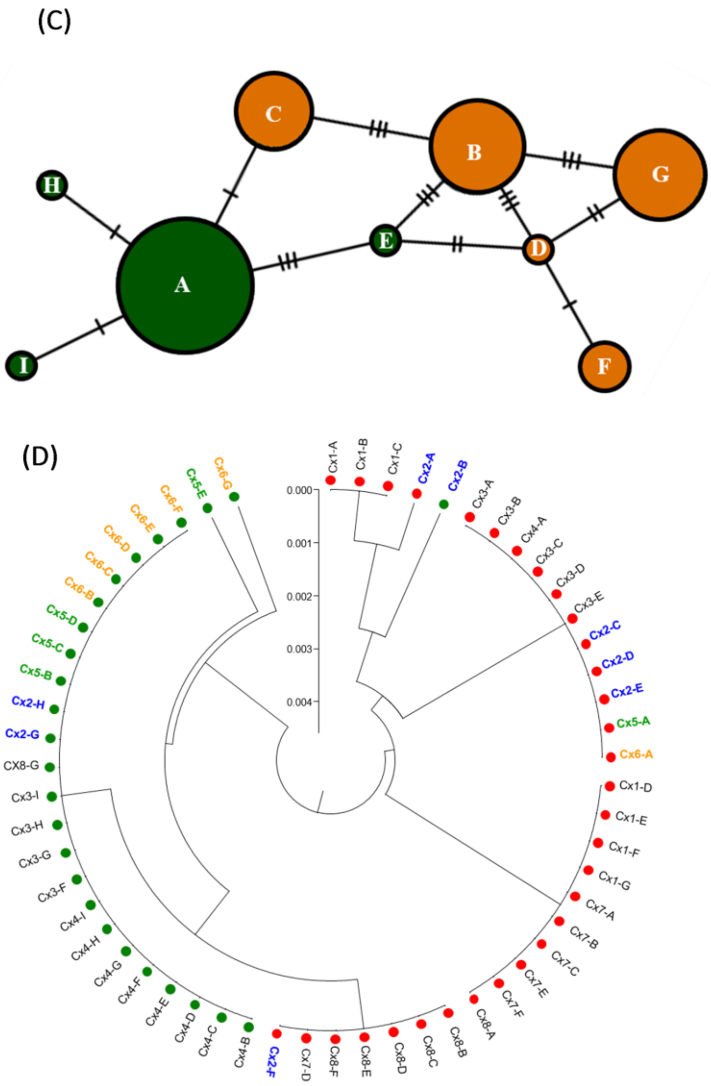


**Figure S3.** Detection of copy number variation in the *Vgsc* and *Ace-1* gene in *Cx. quinquefasciatus*. (A) Allelic *Vgsc-1014* pyrogram showing a heterozygous wild-type/resistant genotype and *Kdr* allelic genotyping with a tri-allelic pattern indicating likely gene duplication due to simultaneous presence of A, C and T alleles, instead of two alleles expected for a diploid genome. (B) Frequency of tri-allelic genotype across Uganda population. (C) Minimum spanning Network built using sequences of a partial fragment of the *Ace-1* gene. Each haplotype is represented by a circle, whose size is proportional to the number of individuals showing that haplotype. Haplotypes are coloured to differentiate haplotypes containing the susceptible allele (Orange) and resistant allele (green). Hatch marks represent mutational steps separating observed haplotypes. (D) Dendrogram constructed using partial sequence of the *Ace*-1 gene from individuals with a likely duplicated haplotype. Green and red dots correspond to susceptible and resistant G119S haplotypes, respectively. Haplotypes are labelled Cx followed by a number corresponding to an individual identifier, and a letter from A to I to identify an individual sequenced colony from that individual. Haplotype labels highlighted in blue, green or orange are individuals with more than two haplotypes simultaneously.
