# Supplementary material for: Local selection in the presence of high levels of gene flow: Evidence of heterogeneous insecticide selection pressure across Ugandan Culex quinquefasciatus populations
Source: PLoS Negl Trop Dis. 2017 Oct 3;11(10):e0005917. doi: 10.1371/journal.pntd.0005917 (PMC5640252; doi:10.1371/journal.pntd.0005917)
Supplement: S4 Fig — (PDF) [file pntd.0005917.s010.pdf]

(A)

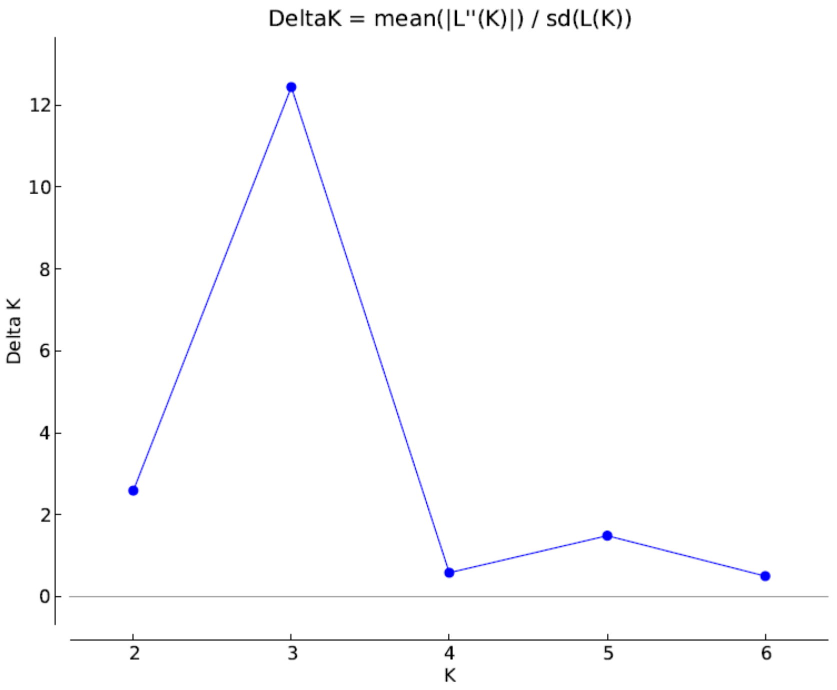

(B)

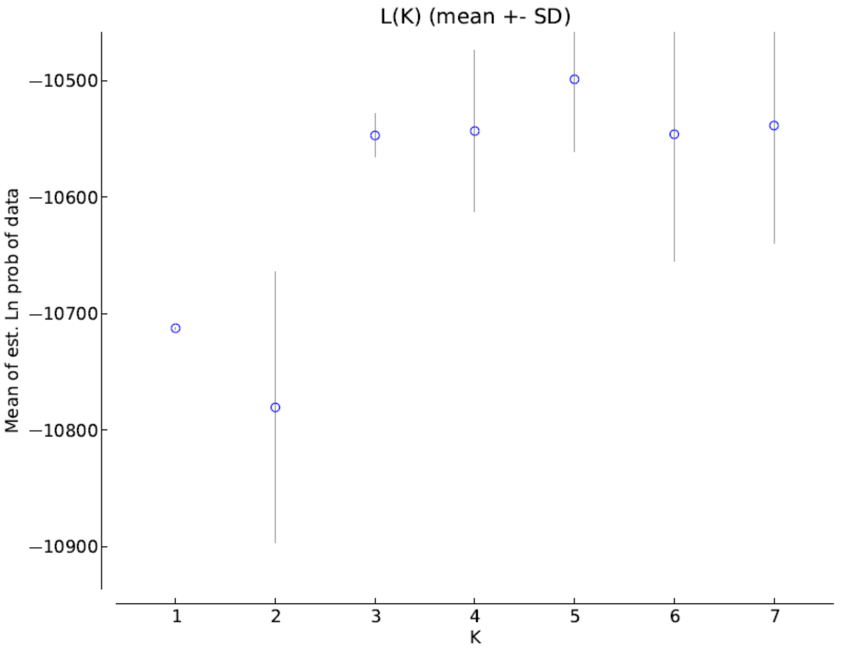

Figure S4. Clustering analysis in Uganda field-collected *Cx. quinquefasciatus* mosquitoes, using Bayesian assignment implemented in the STRUTURE software. (A) Mean likelihood (*L*(*K*) (±s.d.)) over 20 runs dividing the entire dataset into *K* populations, for *K* values between 1 and 7. (B) Delta (*K*) where the modal value of the distribution is considered as the highest level of structuring, in our case three clusters. Graphics depicts in (A) and (B) where constructed using Structure Harvest based on 20 replicated runs of STRUTURE for K values ranging from 1 to 7.
