# Supplementary material for: Local selection in the presence of high levels of gene flow: Evidence of heterogeneous insecticide selection pressure across Ugandan Culex quinquefasciatus populations
Source: PLoS Negl Trop Dis. 2017 Oct 3;11(10):e0005917. doi: 10.1371/journal.pntd.0005917 (PMC5640252; doi:10.1371/journal.pntd.0005917)
Supplement: S5 Fig — (PDF) [file pntd.0005917.s011.pdf]

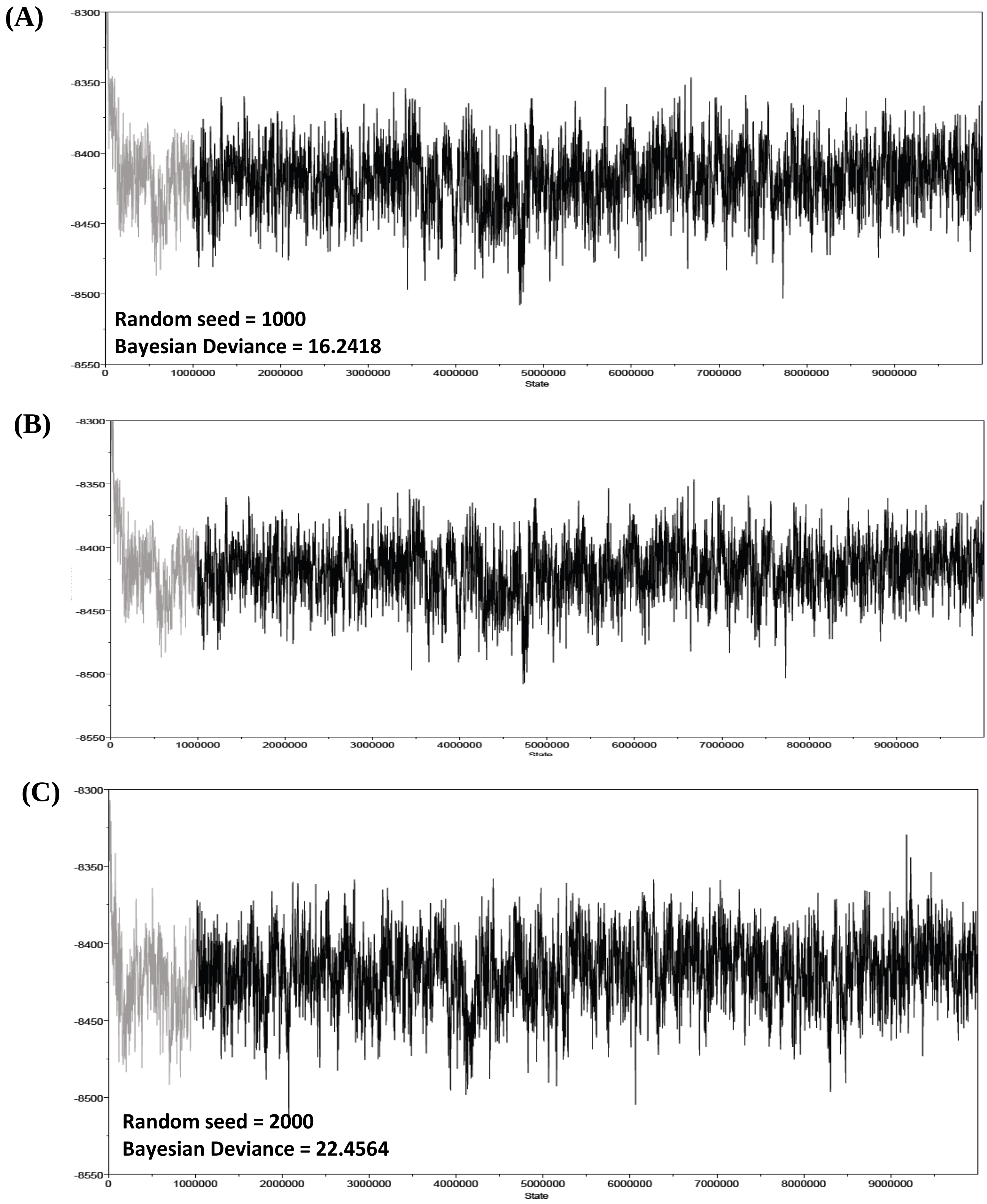

Figure S5 continue

(D)

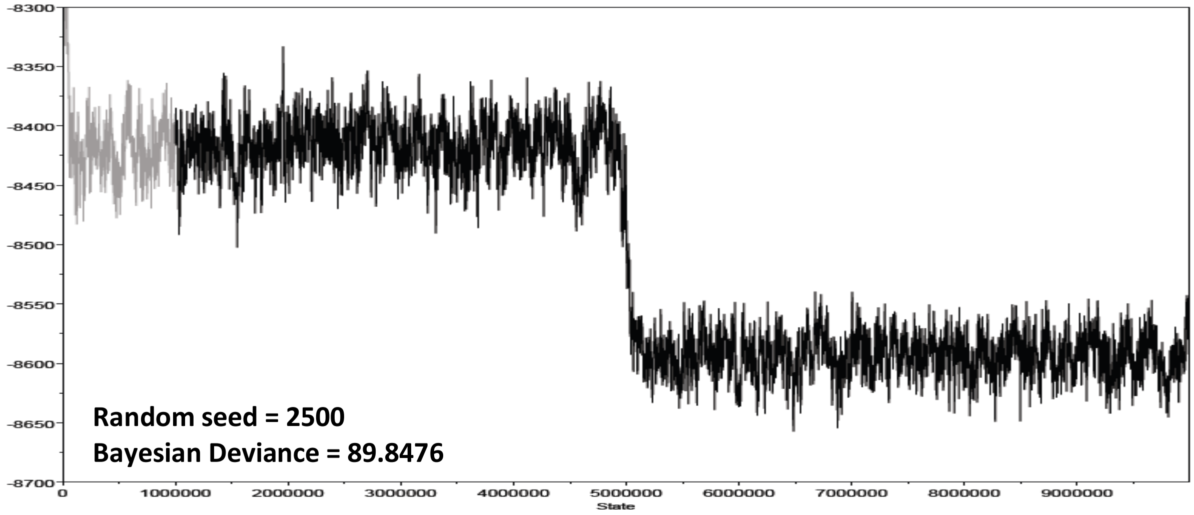

(E)

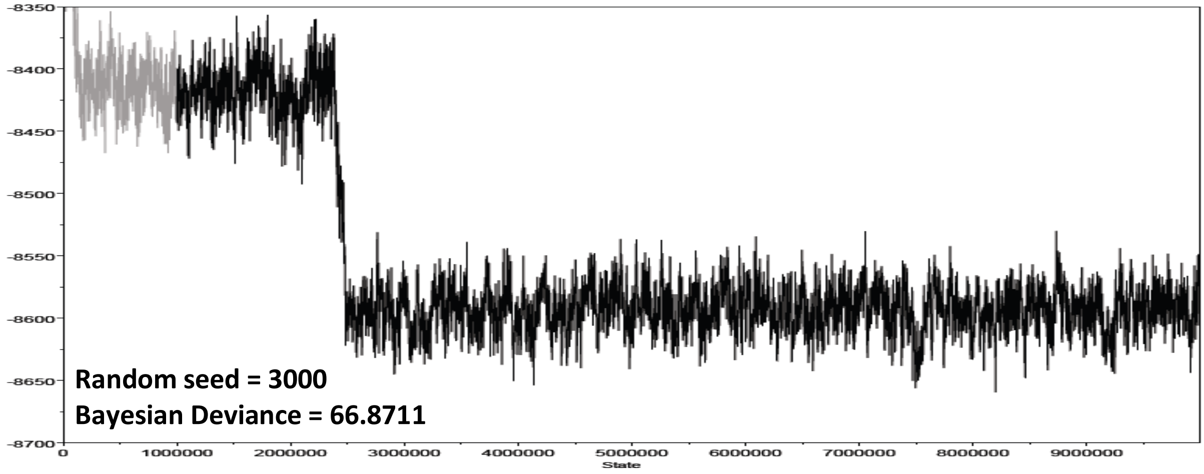

Figure S5 BAYESASS 3.0 Trace-plot and analysis parameters from five parallel runs (A-E) to detect the lowest Bayesian deviance and examine convergence. The X-axis is log probability. The Y-axis is number of Bayesian iterations. The gray shaded trace represents the burn-in. Random. Running was performed for five distinct random seed starting with 1000 and increasing of 500 for each run. In all running the conduction are: MCMC iterations = 10,000,000; burn-in =1,000,000; Sampling interval =2,000; all mixing parameters set as default (0.10), with exception for allele frequencies; DA= 0.3 and inbreed coefficient; DF = 0.3).
