# Supplementary material for: Local selection in the presence of high levels of gene flow: Evidence of heterogeneous insecticide selection pressure across Ugandan Culex quinquefasciatus populations
Source: PLoS Negl Trop Dis. 2017 Oct 3;11(10):e0005917. doi: 10.1371/journal.pntd.0005917 (PMC5640252; doi:10.1371/journal.pntd.0005917)
Supplement: S1 Table — (PDF) [file pntd.0005917.s012.pdf]

S1 Table. Location of field-caught mosquitoes, demographic index of collection sites and main features of vector control intervention in Uganda 2011.

				Control interventions based on Uganda Demographic and Health Survey (UDHS) regions*								
					Percentage of households with at least one mosquito net		Average number of nets per household		Percentage that slept under nets last night		Percentage of Households	
**District**	**Population**	**Pop. Density**	**Urb[a]**	**UDHS[b] - Region**	**ITN[C]**	**LLIN[d]**	**ITN**	**LLIN**	**ITN**	**LLIN**	**with IRS[e] in the past 12 months**	**with at least one ITN and/or IRS in the past 12 months**
Jinja	76,057	694	36.6	Central	57.5	55.5	1.2	1.2	43.8	41.9	5.2	59.2
Kampala	1,507,080	7928	100									
Kanungu	252,144	198	20.3	South-western	58.6	57.6	1.2	1.2	29.5	29	0.6	58.6
Tororo	517,082	433	14	Mid-eastern	56.2	55.4	1.2	1.1	35.1	34.2	2.6	56.7

*Based on Uganda Bureau of Statistics (UBOS)[1]

[a] Urb – Level of urbanization

[b] UDHS – Uganda Demographic and Health Survey

[3]ITN- Insecticide treated mosquito net

[d]LLIN- Long lasting insecticidal net

[e] IRS – Indoor Residual Spraying

[1] Uganda Bureau of Statistics. Uganda Demographic and Health Survey 2011. Kampala, Uganda: Uganda Bureau of Statistics, Maryland: ICF International Inc.2012.
