# Supplementary material for: Local selection in the presence of high levels of gene flow: Evidence of heterogeneous insecticide selection pressure across Ugandan Culex quinquefasciatus populations
Source: PLoS Negl Trop Dis. 2017 Oct 3;11(10):e0005917. doi: 10.1371/journal.pntd.0005917 (PMC5640252; doi:10.1371/journal.pntd.0005917)
Supplement: S2 Table — (PDF) [file pntd.0005917.s013.pdf]

Table S2 Sample sizes and indices of genetic diversity at 26 microsatellite loci for four Ugandan *Cx. quinquefasciatus* populations.

**Location**	**Latitude/longitude coordinates**	**N**	**Ne[a]**	H_E	H_O	F_{IS}	**HW**
Jinja	00° 25'N, 33°12' E	41	423 (210-14320)	0.603 (0.108)	0.541 (0.162)	0.116 *	8[b]
Kampala	00°20'N, 32°30' E	44	203 (142-345)	0.597 (0.107)	0.531 (0.128)	0.123 *	5[b]
Kanungu	00°49'S, 29°44' E	39	69 (57-86)	0.612 (0.159)	0.549 (0.199)	0.115 *	8[b]
Tororo	00°45'N, 34°12' E	38	268(159-782)	0.606 (0.134)	0.511 (0.172)	0.170 *	5[b]

N, Sample size; Ne, Estimate of effective population size; $H_{E,}$ mean expected heterozygosity; $H_{O,}$ mean observed heterozygosity; F_{IS}**,** inbreeding coefficient;
HW, Hardy-Weinberg equilibrium.
[a] Linkage disequilibrium method
[b]Number of loci showing departure from Hardy-Weinberg equilibrium after multiple test correction
*$P > 0.05$
