# Supplementary material for: Local selection in the presence of high levels of gene flow: Evidence of heterogeneous insecticide selection pressure across Ugandan Culex quinquefasciatus populations
Source: PLoS Negl Trop Dis. 2017 Oct 3;11(10):e0005917. doi: 10.1371/journal.pntd.0005917 (PMC5640252; doi:10.1371/journal.pntd.0005917)
Supplement: S3 Table — Polymorphic sites identified in a 535 bp partial fragment (intron 2 and exon 3) of Ace-1. (PDF) [file pntd.0005917.s014.pdf]

Table S3 *Ace-1* haplotype diversity based on sequences obtained from *Cx. quinquefasciatus* mosquitoes sampled in Uganda. Polymorphic sites identified in a 535 bp partial fragment (intron 2 and exon 3) of *Ace-1*.

*

Position	12	33	69	75	87	114	189	277	**355***	462	Total of Variation	Haplotype freq
Consensus	A	Y	R	G	A	T	G	G	G	T		
Haplotype												
A		A	C						A		3	0.397
B		G	T				A				3	0.190
C		A	C								2	0.121
D		G	T	A		C					4	0.0172
E		G	T	A					A		4	0.0172
F		G	T	A	C	C					5	0.0517
G		G	T			C				G	4	0.172
H	G	A	C						A		4	0.0172
I		A	C					T	A		4	0.0172

*Indicates the position of the *Ace*1-G119S mutation in exon 3.
