# Supplementary material for: Local selection in the presence of high levels of gene flow: Evidence of heterogeneous insecticide selection pressure across Ugandan Culex quinquefasciatus populations
Source: PLoS Negl Trop Dis. 2017 Oct 3;11(10):e0005917. doi: 10.1371/journal.pntd.0005917 (PMC5640252; doi:10.1371/journal.pntd.0005917)
Supplement: S4 Table — (PDF) [file pntd.0005917.s015.pdf]

Table S4 Microsatellite locus characterization across all *Cx. quinquefasciatus* populations

Locus	Allele number	*Rs*	*PIC*	H_E	*P*	F_{IS}	F_{IT}	F_{ST}	R_{ST}
MCQ 1	4	4.000	0.418	0.490	0.000	0.485	0.487	0.024	0.010
MCQ 2	3	2.884	0.332	0.405	0.000	0.294	0.293	0.025	0.007
MCQ 3	7	6.910	0.454	0.461	0.000	0.252	0.247	0.049	0.034
MCQ 4	4	3.642	0.544	0.621	0.3184	0.065	0.065	0.011	-0.005
MCQ 5	6	5.640	0.641	0.692	0.000	0.199	0.191	0.016	0.030
MCQ 8	5	4.642	0.604	0.664	0.000	0.288	0.294	0.016	-0.015
MCQ 9	6	5.650	0.665	0.709	0.0339	0.141	0.138	0.026	0.029
MCQ 10	8	7.642	0.652	0.694	0.000	0.404	0.407	0.017	-0.006
MCQ 11	7	6.581	0.493	0.545	0.0535	0.094	0.096	0.045	-0.001
MCQ 13	6	5.910	0.602	0.647	0.1393	0.154	0.153	0.036	0.073
MCQ 16	7	7.000	0.696	0.724	0.0281	0.106	0.103	0.014	-0.003
MCQ 19	9	8.596	0.563	0.594	0.0014	0.046	0.044	0.016	0.038
MCQ 20	3	3.000	0.554	0.623	0.000	0.292	0.290	0.018	0.009
MCQ 21	12	11.14	0.617	0.607	0.000	0.303	0.301	0.072	0.113
MCQ 22	9	8.873	0.688	0.723	0.000	0.214	0.214	0.029	0.080
MCQ 23	5	4.600	0.436	0.513	0.000	0.439	0.436	0.009	0.019
MCQ 24	6	5.983	0.653	0.691	0.1059	0.035	0.035	0.017	-0.004
MCQ 25	5	4.985	0.463	0.546	0.000	0.313	0.310	0.012	-0.005
MCQ 26	10	9.873	0.825	0.830	0.6902	0.086	0.084	0.027	0.030
MCQ 28	4	4.000	0.553	0.606	0.000	0.594	0.596	0.022	0.004
MCQ 29	5	4.909	0.61	0.662	0.0163	0.07	0.069	0.029	-0.009
MCQ 31	6	5.642	0.717	0.741	0.2309	-0.021	-0.023	0.030	-0.004
MCQ 33	4	4.000	0.368	0.424	0.000	0.649	0.662	0.032	-0.010
MCQ 34	8	7.541	0.625	0.680	0.000	0.264	0.271	0.016	0.008
MCQ 36	4	3.642	0.355	0.429	0.2056	0.012	0.013	0.004	-0.003
MCQ 37	4	4.000	0.361	0.376	0.693	0.052	0.054	0.039	0.037
MCQ 39	7	6.634	0.527	0.567	0.8118	-0.035	-0.040	0.012	0.007

MCQ 41	6	5.989	0.651	0.703	0.1518	-0.018	-0.022	0.020	0.018
MCQ 42	8	7.636	0.548	0.560	0.8428	-0.028	-0.030	0.053	-0.003
MCQ 45	8	7.7	0.589	0.630	0.0158	0.025	0.021	0.019	0.001

(*R*s), allelic richness. (PIC), polymorphic Information content. (H_E), expected heterozygosity. (F_{IS}, F_{IT}, F_{ST}) Weir and Cockerham's (1984) F-Statistics. (*P*), probability value using Fisher`s method for Hardy-Weinberg departures.
