# Supplementary material for: Local selection in the presence of high levels of gene flow: Evidence of heterogeneous insecticide selection pressure across Ugandan Culex quinquefasciatus populations
Source: PLoS Negl Trop Dis. 2017 Oct 3;11(10):e0005917. doi: 10.1371/journal.pntd.0005917 (PMC5640252; doi:10.1371/journal.pntd.0005917)
Supplement: S5 Table — (PDF) [file pntd.0005917.s016.pdf]

Table S5 Summary of microsatellite variation in different Ugandan populations of *Cx. quinquefasciatus*

	Location															
	Jinja				Kampala				Kanungu				Tororo			
**Locus**	***A***	H_E	***P***	F_{IS}	***A***	H_E	***P***	F_{IS}	***A***	H_E	***P***	F_{IS}	***A***	H_E	***P***	F_{IS}
MCQ 1	4	0.532	0.000*	0.504	3	0.459	0.002^{NS}	0.415	2	0.347	0.007^{NS}	0.479	4	0.586	0.000*	0.523
MCQ 2	2	0.420	0.717	0.060	3	0.489	0.002^{NS}	0.328	2	0.284	0.052	0.362	2	0.400	0.015^{NS}	0.418
MCQ 3	5	0.537	0.015^{NS}	0.240	6	0.403	0.002^{NS}	0.334	5	0.617	0.000*	0.181	5	0.257	0.545	0.091
MCQ 4	3	0.630	0.647	0.083	3	0.596	0.677	0.020	4	0.635	1.000	-0.036	3	0.589	0.195	0.208
MCQ 5	5	0.655	0.045^{NS}	0.230	6	0.675	0.001*	0.370	5	0.724	0.101	-0.049	5	0.671	0.005^{NS}	0.228
MCQ 8	4	0.679	0.218	0.221	5	0.674	0.132	0.066	4	0.661	0.000*	0.582	4	0.600	0.016^{NS}	0.310
MCQ 9	5	0.716	0.759	0.058	6	0.693	0.046^{NS}	0.190	5	0.661	0.241	-0.061	5	0.728	0.012^{NS}	0.306
MCQ 10	7	0.717	0.000*	0.398	7	0.576	0.134	0.221	7	0.734	0.000*	0.451	8	0.702	0.000*	0.522
MCQ 11	5	0.566	0.614	0.022	5	0.552	0.399	-0.017	6	0.530	0.158	0.093	6	0.502	0.014^{NS}	0.226
MCQ 13	5	0.554	0.398	0.175	5	0.691	0.523	0.123	5	0.719	0.689	0.086	5	0.588	0.241	0.162
MCQ 16	7	0.744	0.457	0.094	6	0.643	0.138	0.127	6	0.706	0.213	0.032	7	0.765	0.176	0.152
MCQ 19	7	0.481	0.890	-0.050	8	0.586	0.003^{NS}	0.119	6	0.660	0.096	-0.230	7	0.616	0.004^{NS}	0.328
MCQ 20	3	0.636	0.513	0.129	3	0.625	0.006	0.355	3	0.566	0.000*	0.499	3	0.626	0.125	0.193
MCQ 21	10	0.461	0.000*	0.414	10	0.741	0.079	0.208	7	0.712	0.023^{NS}	0.163	5	0.474	0.012^{NS}	0.330
MCQ 22	5	0.637	0.013^{NS}	0.359	4	0.611	0.424	0.060	9	0.811	0.062	0.095	8	0.790	0.007*	0.294
MCQ 23	4	0.500	0.000*	0.598	4	0.531	0.000*	0.367	3	0.494	0.000*	0.475	3	0.488	0.064	0.329
MCQ 24	6	0.702	0.004^{NS}	0.108	6	0.715	0.445	-0.069	5	0.702	0.445	-0.010	6	0.609	0.357	0.105
MCQ 25	4	0.508	0.001*	0.386	5	0.554	0.055	0.272	3	0.565	0.365	0.200	4	0.521	0.002^{NS}	0.405

MCQ 26	10	0.839	0.465	-0.033	9	0.826	0.236	0.103	9	0.820	0.315	0.168	10	0.789	0.251	0.046
MCQ 28	4	0.635	0.000*	0.658	4	0.639	0.000*	0.488	4	0.605	0.000*	0.698	4	0.491	0.000*	0.531
MCQ 29	4	0.676	0.645	-0.127	4	0.600	0.090	0.158	5	0.637	0.007[NS]	0.145	4	0.698	0.910	0.060
MCQ 31	5	0.697	0.972	-0.107	5	0.730	0.259	-0.015	6	0.723	0.613	-0.015	5	0.778	0.647	-0.035
MCQ 33	3	0.316	0.000*	0.795	3	0.377	0.001*	0.465	3	0.501	0.000*	0.713	4	0.446	0.000*	0.678
MCQ 34	6	0.708	0.001*	0.409	6	0.588	0.000*	0.271	5	0.662	0.000*	0.115	6	0.708	0.003[NS]	0.285
MCQ 36	3	0.421	0.860	-0.087	2	0.397	1.000	-0.019	3	0.388	0.747	0.086	4	0.488	0.279	0.096
MCQ 37	4	0.483	0.694	0.102	4	0.451	0.249	-0.097	3	0.145	1.000	-0.048	3	0.406	0.631	0.106
MCQ 39	6	0.556	0.245	0.090	5	0.485	1.000	-0.067	5	0.601	0.724	-0.096	7	0.601	0.763	-0.081
MCQ 41	5	0.677	0.707	-0.068	5	0.668	0.288	0.024	4	0.743	0.094	-0.057	4	0.690	0.914	-0.016
MCQ 42	6	0.535	0.869	-0.036	8	0.534	0.558	-0.053	4	0.407	0.676	-0.059	6	0.738	0.655	-0.127
MCQ 45	6	0.658	0.003[NS]	-0.062	7	0.500	0.181	0.056	5	0.682	0.321	0.110	7	0.649	0.006[NS]	-0.041

(*A*), number of alleles. (H_E), expected heterozygosity. (*P*), probability value using Fisher`s method for Hardy-Weinberg departures. (FIS), Weir and Cockerham's (1984) inbreeding coefficient.
