# Supplementary material for: Local selection in the presence of high levels of gene flow: Evidence of heterogeneous insecticide selection pressure across Ugandan Culex quinquefasciatus populations
Source: PLoS Negl Trop Dis. 2017 Oct 3;11(10):e0005917. doi: 10.1371/journal.pntd.0005917 (PMC5640252; doi:10.1371/journal.pntd.0005917)
Supplement: S6 Table — (PDF) [file pntd.0005917.s017.pdf]

Table S6 Pairwise F_{ST} estimates (Weir & Cockerham 1984) across Ugandan *Cx quinquefasciatus* populations

	Jinja	Kampala	Kanungu	Tororo
Jinja				
Kampala	0.0071*			
Kanungu	0.0267*	0.0217*		
Tororo	0.0132*	0.0188*	0.0293*	

*Significant at adjusted nominal level of (5%), with P = 0.008333 for multiple comparisons.
