# Supplementary material for: Local selection in the presence of high levels of gene flow: Evidence of heterogeneous insecticide selection pressure across Ugandan Culex quinquefasciatus populations
Source: PLoS Negl Trop Dis. 2017 Oct 3;11(10):e0005917. doi: 10.1371/journal.pntd.0005917 (PMC5640252; doi:10.1371/journal.pntd.0005917)
Supplement: S7 Table — (PDF) [file pntd.0005917.s018.pdf]

Table S7 Analysis of molecular variance (AMOVA) for four *Cx quinquefasciatus* populations from Uganda, using 26 microsatellite markers.

**Source of Variation**	**d.f.**	**SS**	**MS**	**Variance Component**	**% Total Variance**
Within populations	162	1113.000	6.870	6.870	83
Among populations	3	65.339	21.780	0.154	2
Among individuals	158	1466.877	9.284	1.207	15
